# ILF2 enhances the DNA cytosine deaminase activity of tumor mutator APOBEC3B in multiple myeloma cells

**DOI:** 10.1038/s41598-022-06226-3

**Published:** 2022-02-10

**Authors:** Yasuhiro Kazuma, Kotaro Shirakawa, Yusuke Tashiro, Hiroyuki Yamazaki, Ryosuke Nomura, Yoshihito Horisawa, Suguru Takeuchi, Emani Stanford, Yoshinobu Konishi, Hiroyuki Matsui, Tadahiko Matsumoto, Fumiko Tanabe, Ryo Morishita, Shinji Ito, Akifumi Takaori-Kondo

**Affiliations:** 1 Department of Hematology and Oncology, Graduate School of Medicine, Kyoto University, 54 Shogoin Kawahara-cho, Sakyo-ku, Kyoto, 606-8507 Japan; 2 CellFree Sciences Co., Ltd., Ehime, Japan; 3 Medical Research Support Center, Graduate School of Medicine, Kyoto University, Kyoto, Japan

**Keywords:** Cancer, Cell biology

## Abstract

DNA cytosine deaminase APOBEC3B (A3B) is an endogenous source of mutations in many human cancers, including multiple myeloma. A3B proteins form catalytically inactive high molecular mass (HMM) complexes in nuclei, however, the regulatory mechanisms of A3B deaminase activity in HMM complexes are still unclear. Here, we performed mass spectrometry analysis of A3B-interacting proteins from nuclear extracts of myeloma cell lines and identified 30 putative interacting proteins. These proteins are involved in RNA metabolism, including RNA binding, mRNA splicing, translation, and regulation of gene expression. Except for SAFB, these proteins interact with A3B in an RNA-dependent manner. Most of these interacting proteins are detected in A3B HMM complexes by density gradient sedimentation assays. We focused on two interacting proteins, ILF2 and SAFB. We found that overexpressed ILF2 enhanced the deaminase activity of A3B by 30%, while SAFB did not. Additionally, siRNA-mediated knockdown of ILF2 suppressed A3B deaminase activity by 30% in HEK293T cell lysates. Based on these findings, we conclude that ILF2 can interact with A3B and enhance its deaminase activity in HMM complexes.

## Introduction

Apolipoprotein B mRNA-editing enzyme catalytic polypeptide-like (APOBEC) family proteins have DNA cytidine deaminase activity that restricts retroviruses and retrotransposons by inducing hypermutations and degradation of replication intermediates. APOBECs are endogenous sources of DNA mutations that are often seen in many human cancers, including multiple myeloma^1^. Accumulation of APOBEC signature mutations is correlated with disease progression and poor overall survival in multiple myeloma^2–5^, especially in tumors with translocations between the *IgH* and *c-MAF* (t(14;16)) or *MAFB* (t(14;20)) genes. Among APOBEC3 enzymes, APOBEC3B (A3B) is mainly expressed in the nucleus^6^. We previously reported decreased deaminase activity and fewer APOBEC signature mutations upon shRNA-mediated A3B knockdown in myeloma cells, suggesting that, among APOBECs, A3B plays a major role in cytidine deamination-related mutagenesis in myeloma cells^7^.

Transcriptional and post-transcriptional mechanisms regulate A3B function. The canonical and non-canonical NF-κB pathway and b-Myb enhance A3B transcription, while E2F complexes suppress it^8–11^. Post-transcriptional regulatory mechanisms include protein kinase A mediated phosphorylation which inhibits A3B mutagenic activity^12^ and alternative splicing of A3B which creates non-mutagenic isoforms^13^.

Recent studies have shown that co-factors of A3B can also affect its function. For example, the estrogen receptor (ER) recruits A3B at ER binding regions and introduces C-to-U deamination which facilitates ER target gene expression in breast cancer cells^14^. DHX9 interacts with A3B and inhibits its binding to pregenomic HBV RNA, attenuating the anti-HBV effect of A3B^15^. An Epstein–Barr viral protein, BORF2, interacts with A3B through its catalytic domain and inhibits A3B deaminase activity^16^. Polycomb repressor complex 2 also interacts with A3B and reduces the occupancy of H3K27me3 on promoters of the chemokine CCL2, modulating the microenvironment in hepatocellular carcinoma^17^.

APOBEC3 deaminase activity is strongly inhibited by RNA^18^. Another APOBEC3 protein, APOBEC3G (A3G), forms catalytically inactive high molecular mass (HMM) complexes and RNase A treatment disrupts HMM complexes into catalytically active low molecular mass (LMM) complexes^19–22^. A3B also forms HMM complexes, but RNase A treatment activates A3B without disrupting HMM into LMM complexes^23^. Multiple surface hydrophobic residues in its N-terminal domain regulate the molecular assembly and deaminase activity of A3B^23^. Mutation of these residues severely impairs interaction with multiple heterogeneous nuclear ribonucleoproteins (hnRNPs), which associate with A3B in an RNA-dependent manner^23–25^. These RNA-dependent interacting proteins seem to act as potential regulatory elements of A3B deaminase activity.

We hypothesized that the components of A3B HMM complexes may regulate A3B deaminase activity leading to APOBEC-mediated mutagenesis. In this study, we performed an interactome analysis using myeloma cell lines which express high levels of endogenous A3B. We previously developed myeloma cell lines which have a 3 × FLAG-tag sequence inserted at the C-terminus of the *A3B* gene via CRISPR/Cas9 editing, allowing us to directly analyze endogenous A3B expression using an anti-FLAG antibody^26^. Using these FLAG-knock-in cell lines, we showed that A3B HMM complexes are comprised of multiple RNA-binding proteins or hnRNPs. Among them, interleukin enhancer-binding factor 2 (ILF2) interacts with A3B in an RNA-dependent manner and enhances A3B deaminase activity.

## Results

### Proteomic analysis identifies APOBEC3B-interacting proteins in multiple myeloma cell lines

**Figure 1:**
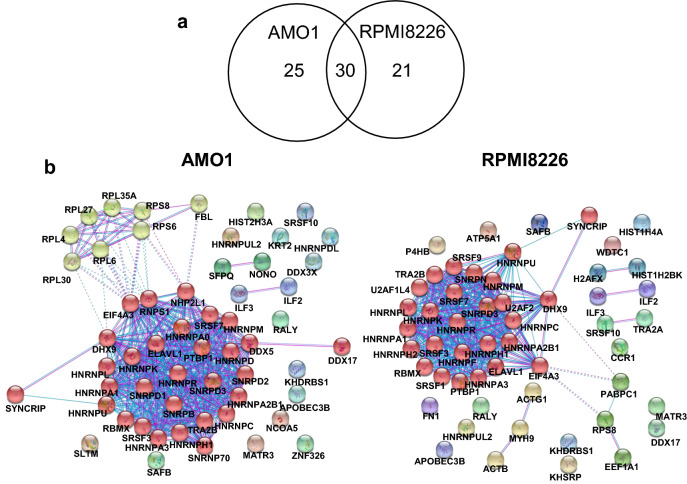
Analysis of the A3B-FLAG interactome in multiple myeloma cell lines. (**a**) Venn diagram of the proteins identified in MS analysis. We identified 55 interacting protein candidates in AMO1 and 51 candidates in RPMI8226 cells. As shown in Supplementary Table 1, 30 protein candidates were identified in both datasets. (**b**) Analysis based on the STRING interaction database showing the putative A3B-interacting proteins identified by MS clustered largely into ribonucleoprotein complexes (*left*, AMO1, *right*, RPMI8226).

We immunoprecipitated (IP) endogenous FLAG-tagged A3B proteins from nuclear extracts of AMO1-A3B-3 × FLAG-IRES-EGFP (AMO1-KI), RPMI8226-A3B-3 × FLAG-IRES-EGFP (RPMI-KI), and wild type cells (AMO1-WT and RPMI-WT) as negative controls^26^ with the anti-FLAG M2 antibody (Supplementary Fig. S1a, b), and analyzed the co-precipitated proteins by mass spectrometry (Supplementary Fig. S2). Mass spectrometry analyses successfully revealed peptides in the FLAG-IP samples (Supplementary Figs. S3, S4). Putative interacting proteins were selected as follows: unique peptide count (95% confidence) ≧2, normalized abundance≧2000 and fold change relative to wild type cell lines ≧2.0 (analyzed with Progenesis QI). We identified 55 interacting protein candidates in AMO1 and 51 candidates in RPMI8226 cells, of which 30 candidates were common in both cell types (Fig. 1a,b; Supplementary Table S1; Supplementary dataset files 1 and 2).

### A3B-interacting protein candidates are clustered largely into ribonucleoprotein complexes

To characterize the A3B-interacting protein candidates obtained by mass spectrometry, we employed the STRING interaction database (http://www.string-db.org/^27^). We extracted the protein–protein interaction profiles of the 30 candidates that were common between AMO1 and RPMI8226 cells. Our analysis revealed that A3B-interacting protein candidates clustered largely into ribonucleoprotein complexes (Fig. 1). GO enrichment analysis demonstrated that a large proportion of them were associated with terms related to RNA metabolism, including RNA binding, mRNA splicing, translation, and regulation of gene expression (Supplementary dataset file 3). KEGG pathway analysis revealed that these interacting protein candidates belong to the spliceosome and ribosome complexes (Supplementary Fig. S1a,b).

### Most of the interacting proteins bind to A3B in an RNA-dependent manner

**Figure 2:**
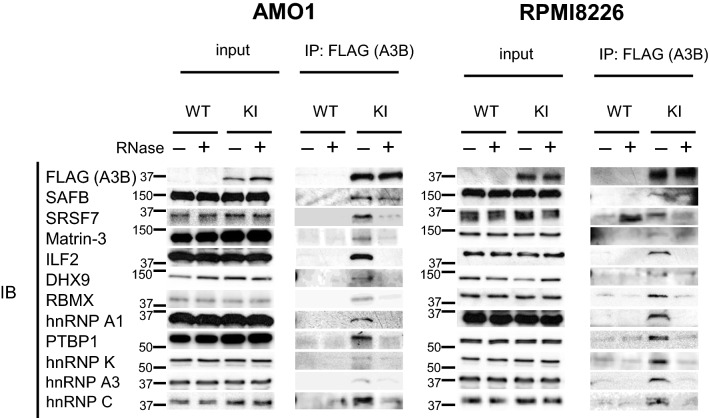
Validation of the interaction with A3B by co immunoprecipitation. Immunoblot analysis of whole cell lysates (3% input) prior to IP and after anti FLAG IP, with or without RNase A treatment (*left*, AMO1, *right*, RPMI8226). All images shown here are representative of three independent experiments. *WT*, wild type (parental) cell *KI*, 3 × FLAG IRES EGFP knock in cell lines. Original blots are presented in Supplementary Figure S13.

We performed co-immunoprecipitation assays in myeloma cell lines to confirm the binding between A3B and the following representative A3B-interacting proteins: SAFB, SRSF7, Matrin-3, interleukin enhancer-binding factor 2 (ILF2), DHX9, RBMX, hnRNP A1, PTBP1, hnRNP K, hnRNP A3 and hnRNP C. Myeloma cell lysates were subjected to co-immunoprecipitation assays using anti-FLAG M2 affinity gel with or without RNase A. The anti-FLAG antibody specifically co-immunoprecipitated the interacting proteins that were detected by immunoblotting (Fig. 2 and Supplementary Fig. S6). We observed that in most cases the interaction was abolished in the presence of RNase A suggesting that the interaction was RNA-dependent. Among the 11 proteins tested in Fig. 2, SAFB was still detected after RNase A treatment, suggesting an RNA-independent interaction with A3B.

### Most of the interacting proteins are components of HMM complexes

**Figure 3:**
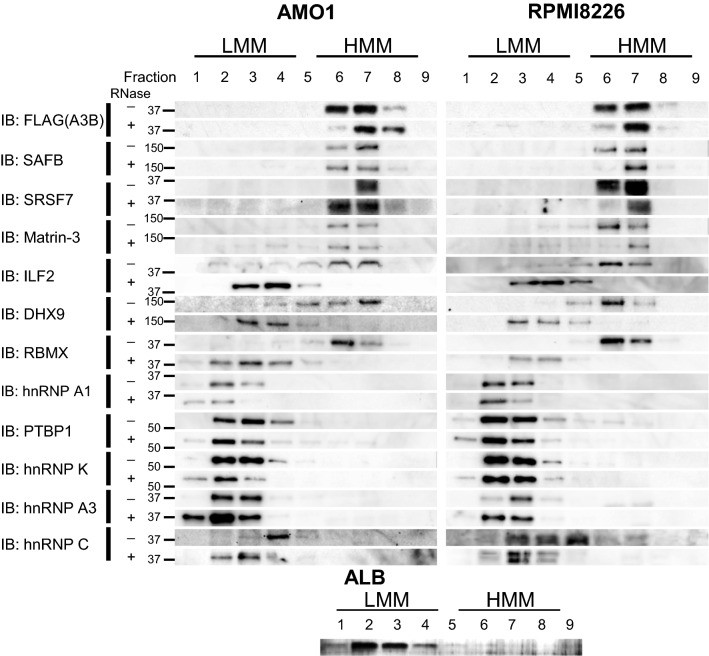
Density gradient sedimentation analysis showing that ILF2 is a component of HMM complexes. Whole cell lysates from AMO1 KI and RPMI8226 KI or lysis buffer containing albumin were loaded on to a discontinuous OptiPrep gradient (4 36%) and centrifuged at 25,000 rpm for 14 h at 4 °C. The separated fractions were analysed by Western blotting with specific antibodies against FLAG and each interactor candidate (*left*, AMO1, *right*, RPMI8226). The position of albumin as a molecular weight marker was confirmed using silver staining. *LMM* Low molecular mass. *HMM* High molecular mass. Original blots are presented in Supplementary Figure S14.

We also tested whether these proteins form HMM complexes with A3B in AMO1-KI and RPMI8226-KI cells by using density gradient sedimentation assays (Fig. 3, Supplementary Figs. S7, S8). We detected SAFB, SRSF7, Matrin-3, ILF2, DHX9, and RBMX in the high-density fractions, but not hnRNP A1, PTBP1, hnRNP K, hnRNP A3, or hnRNP C. After RNase A treatment, most of the putative interacting proteins were observed in the low-density fractions, but not in the high-density fractions. However, SAFB, SRSF7, and Matrin-3 remained in the high-density fractions even after RNase A treatment. Taken together, components of A3B HMM complexes are SAFB, SRSF7, and Matrin-3 via RNA-independent interactions, and ILF2, DHX9, and RBMX via RNA-dependent interactions.

We performed DNA deaminase activity assays with each fraction with or without RNase to evaluate the impact of these interactions on A3B functionality (Supplementary Fig. S9). Consistent with previous reports, RNase treatment increased deaminase activity. Interestingly, higher density fractions showed strongest activity after RNase treatment. It is possible that after RNA depletion, A3B and other DNA-binding proteins form RNA-independent high molecular masses.

### ILF2 co-localizes with A3B in the nucleus and enhances the cytidine deaminase activity of A3B

We further characterized two A3B interacting proteins: SAFB, which is a nuclear matrix protein known to form molecular assemblies^28^, and which interacted with A3B in an RNA-independent manner (shown in Fig. 2), and ILF2, which interacted with A3B in an RNA-dependent manner and which is the only protein shown to contribute to multiple myeloma pathogenesis^29^ among the interacting proteins listed so far.

**Figure 4:**
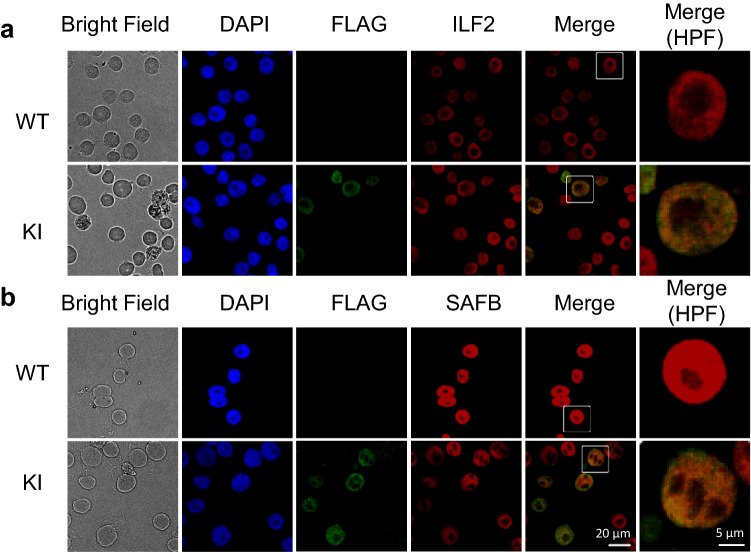
A3B co localizes with ILF2 and SAFB in the nucleus. AMO1 KI cells were subjected to immunofluorescence using Alexa Fluor 555 labelled anti mouse secondary antibody. Endogenous ILF2 (**a**) and SAFB (**b**) were visualized using Alexa Fluor 647 labelled anti rabbit secondary antibody and DAPI was used to stain the nucleus. Image acquisition was performed with a confocal laser scanning microscope (BZ X800, KEYENCE). *WT*, Wild type (parental) cell lines. *KI*, 3 × FLAG IRES EGFP knock in cell lines. *HPF* High power field.

First, we checked the intracellular localization of these proteins in AMO1-KI cells by　immunofluorescence microscopy. We observed that both SAFB and ILF2 localized in the nucleus. Taking into consideration also the results of co-IP and density gradient sedimentation analyses, these proteins co-localize with A3B in the nucleus (Fig. 4a,b). We confirmed the interactions in the nucleus using co-IP and reciprocal co-IP with nuclear lysates from HEK293T cells (Supplementary Fig. S10a,b).

Second, to examine whether these interacting proteins affect A3B function, we measured the deaminase activity of A3B in vitro using purified proteins produced in a wheat germ cell-free expression system (Supplementary Fig. S11a–d). A3B-C terminal domain (CTD), ILF2 and oligonucleotides were mixed and rotated for 30 min at 37 °C. We used the fluorescent protein Venus as a negative control because it doesn’t interact with A3B. ILF2 enhanced A3B deaminase activity compared to Venus in a dose-dependent manner (Supplementary Fig. S12a,b). We could not test SAFB in this assay because we were not able to purify in vitro-translated SAFB proteins by a standard method.

**Figure 5:**
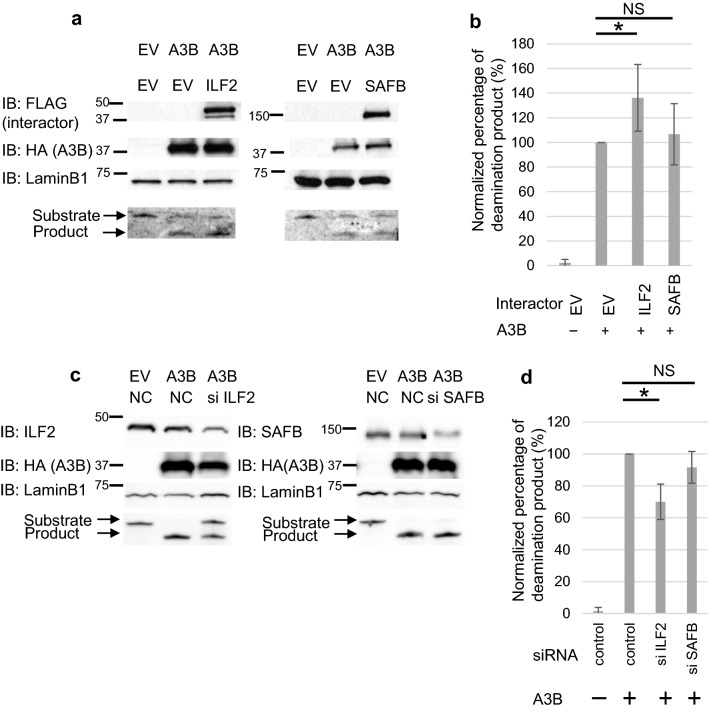
ILF2 enhances A3B cytidine deaminase activity. (**a**) Immunoblot analysis of interactor overexpressing HEK293T cell lysate (upper panel) and representative TBE urea PAGE analysis of A3B cytidine deaminase activity (lower panel). LaminB1 is an endogenous control. (**b**) Quantification of the cytidine deaminase activity data in five biologically independent experiments. Deaminated/total (deaminated and aminated) percentage of product was calculated and normalized by the control sample overexpressing A3B without interactors. (**c**) Immunoblot analysis of interactor knocked down HEK293T cell lysate and representative TBE urea PAGE analysis of A3B cytidine deaminase activity. (**d**) Quantification of the cytidine deaminase activity data in five biologically independent experiments. Deaminated/total (deaminated and aminated) percentage of product was calculated and normalized by the sample overexpressing A3B with control siRNA. Values are means standard deviations (error bar). Asterisks (*) show statistically significant difference *p* < 0.05). *EV* Empty vector, *NC* Negative control (scramble siRNA). Original blots and gels are presented in Supplementary Figure S15.

Third, we measured the deaminase activity of A3B using HEK293T cell lysates with overexpressed A3B and interacting proteins as previously described^12^. Overexpression of ILF2 enhanced the deaminase activity of A3B by 30%, but of SAFB did not (Fig. 5a,b). Additionally, siRNA-mediated knockdown of ILF2 suppressed A3B deaminase activity by 30% (Fig. 5c,d). Taken together, these data indicate that ILF2 positively regulates the deaminase activity of A3B.

## Discussion

RNA-mediated formation of HMM ribonucleoprotein complexes plays a crucial role in APOBEC deaminase activity. Mishra et al. reported that mass spectrometry-based proteomics using HEK293T cells with overexpressed A3B identified A3B-interacting proteins, however, the precise role of these proteins has not been elucidated^24^. In this study, we have performed for the first time a proteomic analysis of the A3B interactome in a multiple myeloma context. We used myeloma cells that endogenously express A3B and identified 30 putative interacting proteins. Most of the putative interacting proteins were RNA-binding proteins including hnRNPs, ribosomal proteins and splicing factors. Co-IP experiments showed that, except for SAFB, the interactions of these proteins with A3B were RNA-dependent. We also showed that these interacting proteins exist in HMM complexes and most of them move to low-molecular fractions when treated with RNase A. Additionally, immunofluorescence microscopy studies showed that ILF2 and SAFB co-localize with A3B in the nucleus. Lastly, functional assays showed that ILF2 enhanced the deaminase activity of A3B.

Our results indicate that the components of A3B HMM complexes may play important roles in regulating its deaminase activity. In terms of other APOBEC family proteins, components of the complexes they are part of have been reported to modulate their catalytic activity. For example, activation-induced cytidine deaminase (AID) monomers interact with hnRNP K, whereas AID dimers interact with hnRNP L^30^. hnRNP K is involved in DNA cleavage events associated with somatic hypermutation, while hnRNP L is involved in DNA recombination events associated with class switch recombination^31^, suggesting that different interacting proteins play different roles in the function of APOBEC-protein complexes. However, little is known about how protein–protein interactions affect the deaminase activity of APOBEC-protein complexes. Four tyrosine mutations and W127A in the N-terminal domain of A3B impair HMM assembly and A3B deaminase activity^23^. Other components of the HMM complexes also bind to ssDNA and might provide substrates to adjacent A3B, facilitating deamination. RNase A treatment of the complexes drastically elevates deaminase activity^18,23^ although it disrupts RNA-dependent interaction with activating co-factors including ILF2. Additionally, our density sedimentation analysis showed that the fraction with the highest deaminase activity shifted to higher molecular weight fractions after RNase treatment. It is possible that RNA-mediated A3B inhibition might be more important than co-factor mediated activation. Another possibility is that A3B HMM may be converted into RNase-resistant complexes with unknown activating co-factors.

ILF2 is overexpressed in various tumor types, including multiple myeloma^29^. Of note, *ILF2* is located in the chromosomal region 1q21. 1q21 amplification occurs in approximately 30 to 50% of newly diagnosed and 50 to 80% of relapsed/refractory multiple myeloma cases, and is associated with disease progression and drug resistance^32–36^. The candidate genes which participate in the pathogenicity of 1q21 amplification include *MUC1*^37^, *MCL1*^38^, *PDZK1*^39^, *IL6R*^40^, *BCL9*^41^, *CKS1B*^42^, *PSMD4*^43^ and *ILF2*^29^, but the crucial driver oncogenes have not been identified yet. ILF2 induces drug resistance in myeloma cells via mRNA processing and the stabilization of transcripts involved in homologous recombination in response to DNA damage^29^. In this study, we found that ILF2 interacts with A3B and enhances A3B deaminase activity, suggesting that ILF2 may contribute to clonal evolution or drug resistance not only by enhancing mRNA splicing of DNA damage response proteins, but also by increasing A3B deaminase activity.

In summary, we demonstrate that, in myeloma cells, A3B HMM complexes are comprised of multiple RNA-binding proteins and some of them can affect A3B deaminase activity. However, it remains to be established whether these interacting proteins regulate A3B deaminase activity in myeloma cells in vivo. It is possible that other co-factors interact with A3B and play a different role in certain conditions such as under replication stress or after viral infection. Further investigation is required to elucidate which interactions are meaningful in physiological settings.

## Methods

### Cell lines and cell culture

Two human myeloma cell lines, AMO1 and RPMI8226 cells were maintained in RPMI1640 (Nacalai Tesque) containing 20% FBS and 1% PSG (Invitrogen). HEK293T cells were maintained in DMEM (Nacalai Tesque) containing 10% FBS and 1% PSG. A3B reporter cell lines that contain a 3 × FLAG tag at the C-terminus of the *A3B* gene: AMO1-KI and RPMI8226-KI cells were generated as previously reported^26^.

### Co-immunoprecipitation assays for mass spectrometry analysis

For co-immunoprecipitation with an anti-FLAG antibody for mass spectrometry analysis, nuclear extracts were prepared as previously described^44^. 5 × 10^7^ cells were briefly washed once with phosphate-buffered saline and the cell pellet were suspended in Hypobuffer (10 mM HEPES–KOH pH 7.9, 10 mM KCl). After incubating for 15 min on ice, TritonX-100 was added. The lysed cellular suspension was briefly vortexed and microcentrifuged for 30 min at 4 °C. The supernatant was discarded as cytoplasmic extract. The pellet was resuspended in high salt radioimmunoprecipitation (RIPA) buffer (50 mM Tris–HCl pH 8.0, 450 mM NaCl, 1% (v/v) Triton X-100, 0.1% (v/v) SDS, 0.1% (v/v) sodium deoxycholate) and agitated for 30 min at 4° C. After centrifugation, the supernatant was mixed with 2 volumes of no salt RIPA buffer (50 mM Tris–HCl pH 8.0, 1% (v/v) Triton X-100, 0.1% (v/v) SDS, 0.1% (v/v) sodium deoxycholate, 5% Glycerol). After pre-clearing with 10 μL of FG beads (Tamagawa Seiki), the supernatant was incubated with antibody-coupled FG beads for 2 h at 4° C. FG beads were conjugated with the anti-FLAG antibody according to the manufacturer’s instructions and then used for immunoprecipitation. The beads were washed five times with low salt RIPA buffer (50 mM Tris–HCl pH 8.0, 150 mM NaCl, 1% (v/v) Triton X-100, 0.1% (v/v) SDS, 0.1% (v/v) sodium deoxycholate) and then incubated for 30 min after adding FLAG elution buffer (TBS supplemented with 150 μg/mL of 3 × FLAG peptide). The protease inhibitor cocktails (Nacalai Tesque) and PhosSTOP (Roche) were added to Hypobuffer and RIPA buffer just before use. The eluted samples were analyzed by silver staining using the Silver Staining MS kit (Wako, Osaka, Japan) according to the manufacturer’s instructions.

### Mass spectrometry

The eluted proteins were precipitated with cold acetone, dried and resuspended in 8 M urea/30 mM ammonium bicarbonate. After reduction and alkylation with dithiothreitol and iodoacetamide, the proteins were digested with trypsin overnight, then purified using Pierce C18 spin columns (Thermo Fisher Scientific), dried and resuspended in 0.1% formic acid. The separation was carried out using Nano-LC-Ultra 2D-plus equipped with cHiPLC Nanoflex (Eksigent, Dublin, CA, USA) in trap-and-elute mode, with trap column (200 μm × 0.5 mm ChromXP C18-CL 3 μm 120 Å (Eksigent)) and analytical column (75 μm × 15 cm ChromXP C18-CL 3 μm 120 Å (Eksigent)). The binary gradients used for the separation were as follows: A98%/B2% to A66.8%/B33.2% in 125 min, A66.8%/B33.2% to A2%/B98% in 2 min, A2%/B98% for 5 min, A2%/B98% to A98%/B2% in 0.1 min, and A98%/B2% for 17.9 min, in which 0.1% formic acid/water and 0.1% formic acid/acetonitrile were used as solvents A and B respectively. The flow rate was 300 nL/min. The analytical column temperature was set to 40 °C. The eluates were infused on-line to a mass spectrometer (TripleTOF 5600 + System with NanoSpray III source and heated interface (SCIEX, Framingham, MA, USA)) and ionized in an electrospray ionization-positive mode. Data acquisition was carried out with an information-dependent acquisition method.

### Label-free quantification of the relative protein abundance

The acquired datasets were analyzed using ProteinPilot 5.0.1 (SCIEX) with the UniProtKB/Swiss-Prot human database (May 2018) appended with known common contaminants (SCIEX). The quality of the database search was confirmed by the false discovery rate analysis in which the reversed amino acid sequences were used as decoy. The reliability of protein identifications was evaluated by the number of identified peptides with confidence of at least 95%, and Unused ProtScores that were calculated by the Pro Group algorithm (SCIEX). Relative abundances of the identified proteins were estimated on the platform of Progenesis QI for Proteomics 4.1 (Nonlinear Dynamics, Newcastle upon Tyne, UK). All raw data files in wiff format (SCIEX) were imported to generate aggregates, and the peptide identification results by ProteinPilot with confidence of at least 95% were used for assignment. Label-free quantification of proteins was performed by relative quantitation using the Hi-N method (Nonlinear Dynamics).

### Functional classification and network of interactions

Protein–protein interaction networks were constructed with the STRING database^27^. GO annotation and KEGG pathway enrichment analysis was conducted using DAVID Functional Annotation Tool^45–49^.

### Antibodies

Antibodies used for protein analysis were as follows: Anti-FLAG M2 antibody (F3165 Sigma-Aldrich), Anti-ILF2 antibody (ab113205 Abcam), Anti-SAFB antibody [EPR13588] (ab187650 Abcam), Anti-RNA Helicase A antibody (ab26271 Abcam), Anti-HNRPA3/HNRNPA3 antibody (ab78300 Abcam), Anti-Matrin-3 antibody [EPR10635(B)] (ab151714 Abcam), Anti-SFRS7/SRSF7 antibody (ab138022 Abcam), hnRNP C1/C2 (D6S3N) Rabbit mAb (#91,327 Cell Signaling Technology), RBMX/hnRNP G (D7C2V Cell Signaling Technology) Rabbit mAb (#14,794 Cell Signaling Technology), PTBP1 (E4I3Q) Rabbit mAb (#57,246 Cell Signaling Technology), hnRNP K (R332) Antibody (#4675 Cell Signaling Technology), Anti-hnRNP A1 Antibody (4B10) (sc-32301 Santa Cruz Biotechnology), Anti-Histone H3 Antibody, CT, pan (07–690 Sigma-Aldrich), Anti-Lamin B1 antibody [EPR8985(B)] (ab133741 Abcam). The primary antibodies were detected using Rabbit IgG HRP Linked Whole Ab (NA934 Cytiva) or Mouse IgG HRP Linked Whole Ab (NA931 Cytiva). The bands were visualized using Pierce ECL Plus Western Blotting Substrate (Thermo Fisher Scientific).

### Co-immunoprecipitation assays for immunoblotting

Myeloma cells were lysed with low salt RIPA buffer containing complete Protease Inhibitor Cocktail (Roche) and PhosSTOP (Roche). The lysates were immunoprecipitated using the ANTI-FLAG M2 Affinity Gel (A2220 Sigma-Aldrich) at 4 °C for 2 h in the presence or absence of RNase A, DNase-free, followed by immunoblotting.

HEK293T cells were transfected with expression vectors for A3B-FLAG using the XtremeGENE HP DNA Transfection Reagent. The nuclear extract was collected as described for the sample preparation for mass spectrometry and immunoprecipitated using the anti-FLAG, anti-ILF2 or anti-SAFB antibody along with Protein G beads at 4 °C, followed by immunoblotting.

### Density gradient sedimentation

Nine layers of 4 to 36% sucrose were prepared in GST lysis buffer (150 mM NaCl, 25 mM HEPES pH 7.4, 0.5% Triton-X100, 1 mM MgCl_2_, 1 mM ZnCl_2_, 10% Glycerol). The whole cell extract, which was made with GST lysis buffer containing protease inhibitor cocktail (Roche), was loaded on top of the gradient and ultracentrifuged for 14 h at 25,000 rpm (CP65; Hitachi Koki) in a SW41Ti rotor. After ultracentrifugation, 10 fractions were collected from the top of the gradient and subjected to immunoblotting and gel-based cytidine deaminase activity assay.

### Immunofluorescence assays

Myeloma cells were air-dried and fixed in 3.7% formaldehyde in phosphate-buffered saline (PBS) for 10 min at room temperature on glass slides using Shandon cytospin 2 (Thermo Fisher Scientific). Fixed cells were permeabilized, reduced and denatured for 30 min at room temperature in PBS buffer containing 0.5% SDS, 5% β-mercaptoethanol and 10% FBS. Then, cells were washed three times with PBS containing 4% FBS and 0.1% Triton X-100 (PET buffer) and incubated with the anti-FLAG antibody (1:200 dilution) and either rabbit anti-ILF2 (1:200 dilution), or anti-SAFB (1:200 dilution) antibody for 1 h. Cells were then washed three times with PET buffer and incubated with Alexa Fluor 555 and Alexa Fluor 647 labeled secondary antibodies (A11078; A11037 Cell Signaling Technology) for 1 h in the dark. All antibodies were diluted with 3% BSA and 0.5% Tween in PBS. Slides were mounted in VECTASHIELD with DAPI (Vector Labs) and observed with a confocal laser scanning microscope (BZ-X800, KEYENCE).

### Knockdown experiments

Transfection and co-transfection were carried out using Lipofectamine RNAiMAX (Invitrogen) according to the manufacturer’s instructions. Cells were harvested 72 h after transfection. Short interfering RNA (siRNA) duplexes in this study were purchased from Thermo Fisher Scientific (ILF2: Silencer select pre-Designed siRNA s7400, s7399; SAFB: Silencer select pre-Designed siRNA s12452, s12453; non-target control: Silencer Select Negative Control #1 siRNA (4,390,843)).

### Protein expression and purification using a wheat germ cell-free expression system

His-tagged A3B (C-terminal domain) was expressed in a wheat germ cell-free expression system using the WEPRO7240H Expression Kit (CellFree Sciences, Matsuyama, Japan). Brij-35 at a final concentration of 0.04% and zinc acetate at a final concentration of 1 µM were added to the translation reaction solution. Expressed proteins were purified by affinity chromatography with nickel–Sepharose.

Flag/GST-tagged ILF2, hnRNP A1, and Venus were expressed in a wheat germ cell-free expression system using the WEPRO7240G Expression Kit (CellFree Sciences, Matsuyama, Japan). Brij-35 at a final concentration of 0.04% was added to the translation reaction solution. Expressed proteins were purified by affinity chromatography with glutathione–Sepharose.

### Gel-based cytidine deaminase activity assays

To prepare cell lysates with overexpressed A3B and interacting proteins, HEK293T cells were transfected with expression vectors for A3B and for each interactor using XtremeGENE HP DNA Transfection Reagent (Roche). The cells were harvested 48 h later, lysed with 120 µl of GST lysis buffer/well and used for gel-based cytidine deaminase activity (CDA) assays. To prepare interactor-depleted lysates, HEK293T cells (5 × 10^5^) were transfected with 60 nM interactor-specific or scrambled siRNA. At 24 h, the cells were transfected with expression vectors for A3B. At 72 h, the cells were harvested, lysed and assayed. 5 µl of HEK293T cell lysate and 1 pmol of single-stranded DNA oligonucleotide (ATTATTATTATTCAAATGGATTTATTTATTTATTTATTTATTT) with 5’-attached FAM were incubated with 0.005 units of UDG and 3.75 µl of reaction buffer in 10 µl reaction volume for 2 h at 37 °C. Subsequently, the oligo products were incubated in 100 mM NaOH for 30 min at 37 °C, stained with loading dye, and denatured, followed by electrophoresis in 20% Tris/urea-acrylamide gel, which was then visualized with Image Quant LAS 500. Purified A3B-CTD, ILF2, and Venus were produced using the wheat germ cell-free expression system^50,51^ and were provided by Cell Free Sciences (Supplementary Fig. S11a–d). For the gel-based CDA assays using purified proteins, we used purified A3B-CTD instead of lysate. Before the reaction, 1 pmol of A3B-CTD and 2, 4, or 8 pmol of each interactor were mixed and rotated for 30 min at 37 °C. For the assays using the gradient fractions from myeloma cell lines, each lysate fraction, DNA substrates and UDG were mixed and incubated for 6 h at 37 °C before the assay.

### Statistical analysis

The signal intensity of the bands in Western blot membranes or Urea-PAGE gels used in CDA assays was quantified using ImageJ version 1.51d (https://imagej.nih.gov/ij/). P values were calculated by paired T test, and *p* < 0.05 was defined as significant difference.

## Supplementary Information

Supplementary Information 1.

Supplementary Information 2.

Supplementary Information 3.

Supplementary Information 4.

## Abbreviations

APOBEC3B: Apolipoprotein B mRNA editing enzyme catalytic subunit-like protein 3B
co-IP: Co-immunoprecipitation
siRNA: Small interfering RNA
ssDNA: Single-stranded DNA
