# Supplementary material for: ILF2 enhances the DNA cytosine deaminase activity of tumor mutator APOBEC3B in multiple myeloma cells
Source: Sci Rep. 2022 Feb 10;12:2278. doi: 10.1038/s41598-022-06226-3 (PMC8831623; doi:10.1038/s41598-022-06226-3)
Supplement: Supplementary file 4 — Supplementary Information 4. [file 41598_2022_6226_MOESM4_ESM.pdf]

Supplementary Information for

ILF2 enhances the DNA cytosine deaminase activity of tumor mutator APOBEC3B in multiple myeloma cells

Yasuhiro Kazuma[1], Kotaro Shirakawa[1]*, Yusuke Tashiro[1], Hiroyuki Yamazaki[1], Ryosuke Nomura[1], Yoshihito Horisawa[1], Suguru Takeuchi[1], Emani Stanford[1], Yoshinobu Konishi[1], Hiroyuki Matsui[1], Tadahiko Matsumoto[1], Fumiko Tanabe[2], Ryo Morishita[2], Shinji Ito[3] and Akifumi Takaori-Kondo[1]

From the [1]Department of Hematology and Oncology, Graduate School of Medicine, Kyoto University, Kyoto, Japan, [2]CellFree Sciences Co., Ltd., Ehime, Japan, [3]Medical Research Support Center, Graduate School of Medicine, Kyoto University, Kyoto, Japan

Supplementary Figure S1

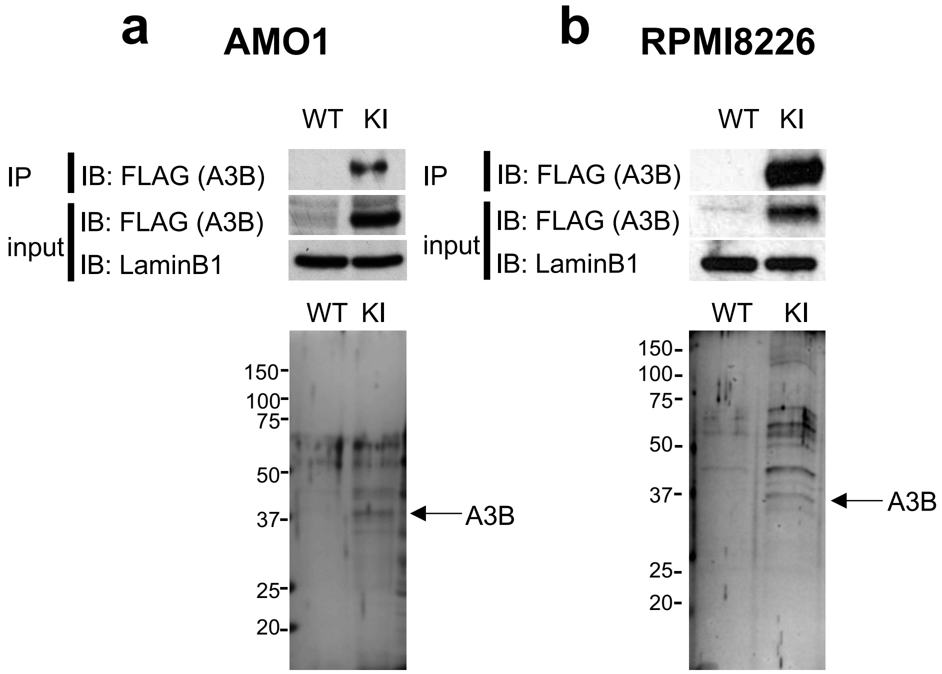

Supplementary Figure S1. Silver-stained gel illustrating co-immunoprecipitated proteins used in MS analysis. Immunoblot analysis (upper panels) and silver staining (lower panels) of the proteins co-immunoprecipitated with A3B-FLAG in AMO1 (a) and RPMI8226 (b) cells. LaminB1 is an endogenous control. *WT*, wild type (parental) cell lines. *KI*, 3 × FLAG–IRES–EGFP knock-in cell lines. Original blots and gels are presented in Supplementary Figure S16.

Supplementary Figure S2

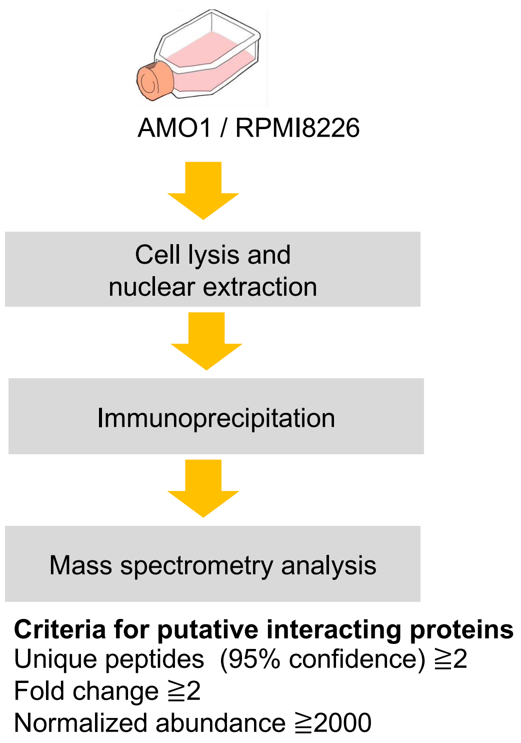

Supplementary Figure S2. Schematic workflow of the interactome analysis. Fold change was calculated as the normalized abundances in knock-in cell lines divided by those in wild type (parental) cell lines.

Supplementary Figure S3

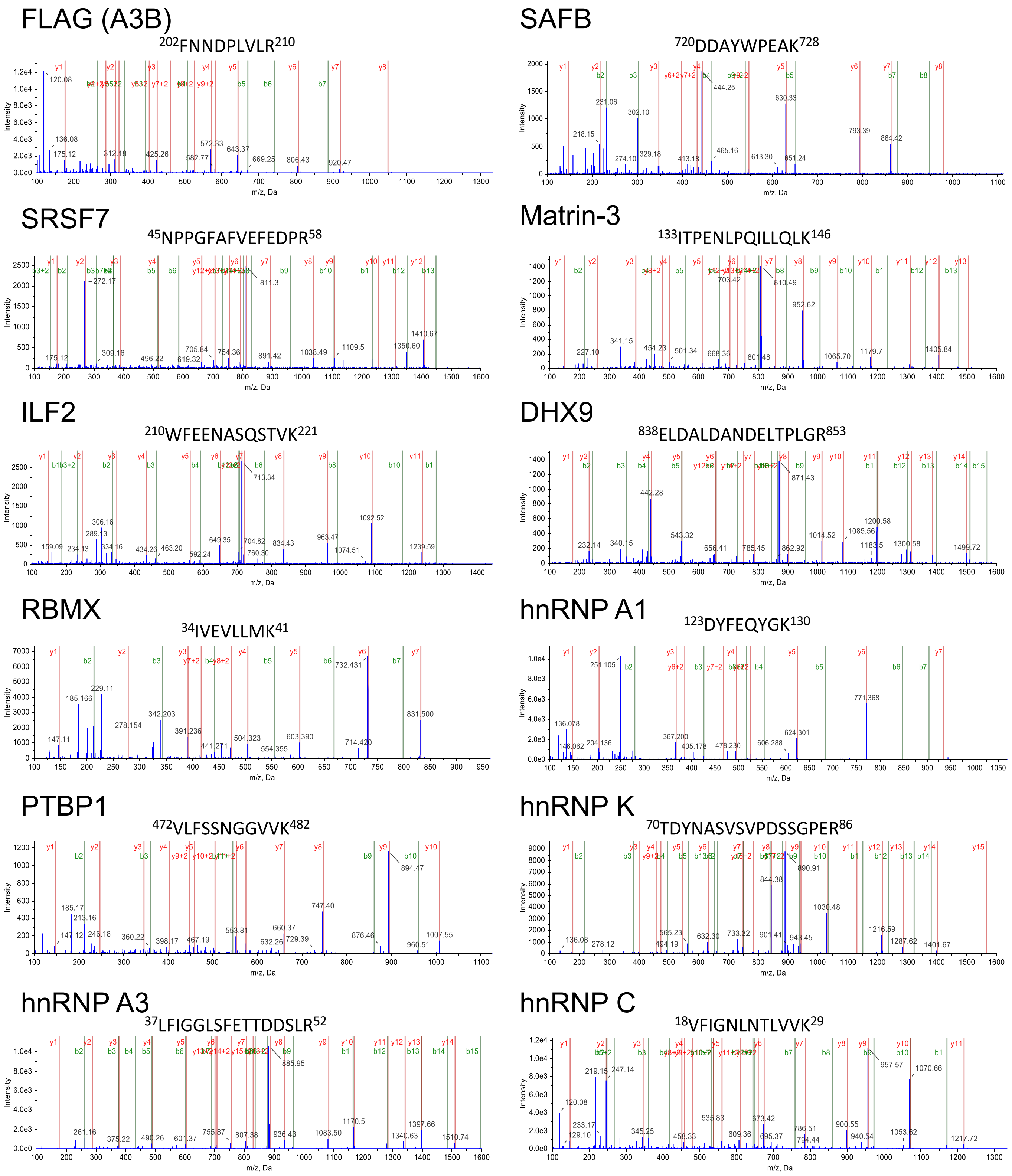

Supplementary Figure S3. Representative spectra of MS/MS showing the identified sequence of peptides derived from representative interacting protein candidates in the FLAG-IP lysate of AMO1 cells**.**

Supplementary Figure S4

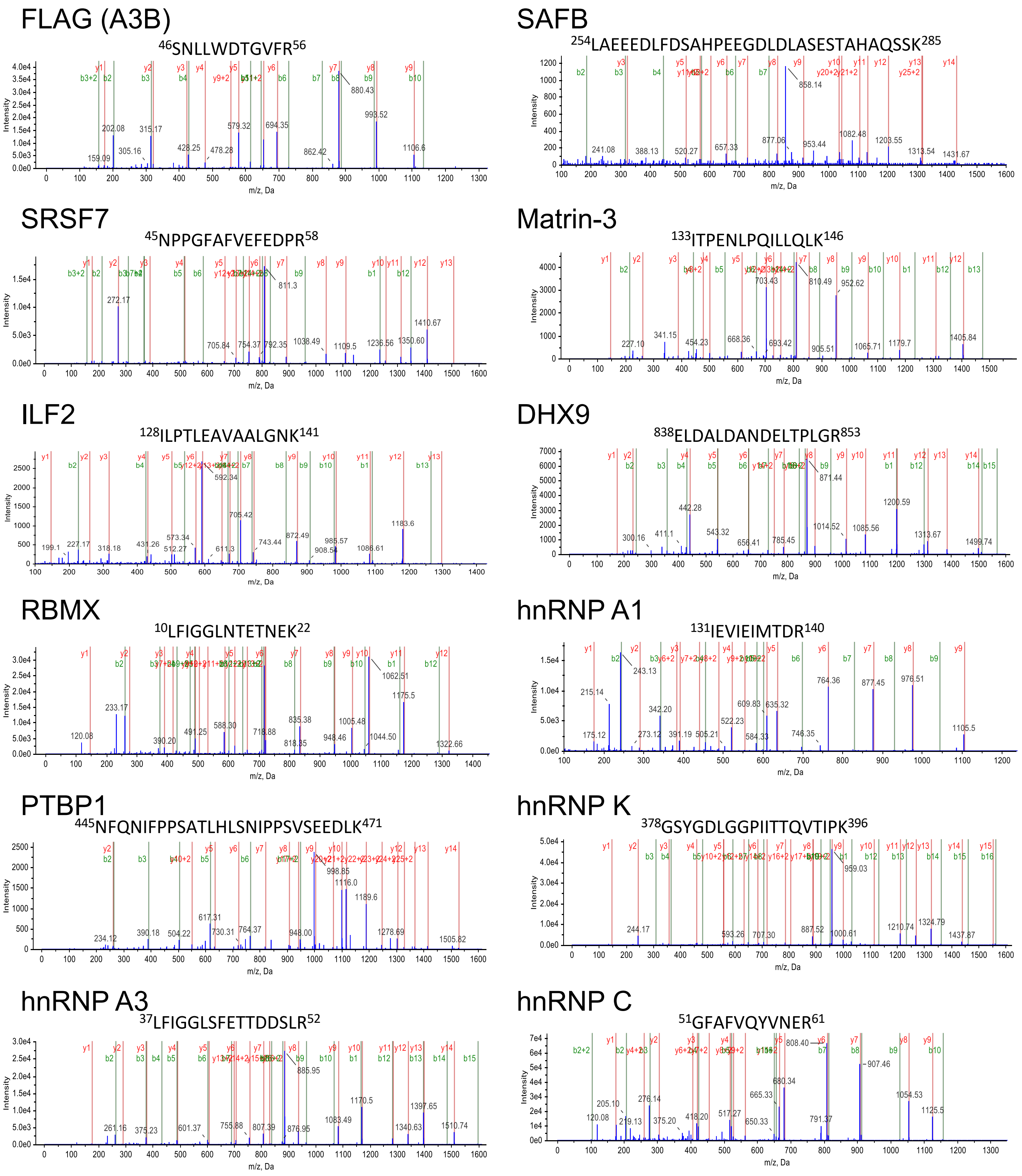

Supplementary Figure S4. Representative spectra of MS/MS showing the identified sequence of peptides derived from representative interacting protein candidates in the FLAG-IP lysate of RPMI8226 cells.

Supplementary Table S1

		AMO1				RPMI8226			
			Abundance				Abundance		
GENEPEPT ID	Protein Name	Unique peptides	AMO1-KI	AMO1-WT	Fold Change	Unique peptides	RPMI-KI	RPMI-WT	Fold Cchange
Q9UH17	APOBEC3B	54	32347.2	226.6	142.7	30	28176.5	90.8	310.4
**RNA helicase**									
Q92841	DDX17	23	6295.8	1132.9	5.6	21	5288.4	56.2	94.1
Q08211	DHX9	45	2526.1	387.9	6.5	39	4687.1	109.7	42.7
P38919	EIF4A3	21	2835.2	985.8	2.9	7	2790.7	52.3	53.3
**Ribonucleoproteins**									
P09651	HNRNPA1	27	28337.5	700.2	40.5	41	72566.6	288.1	251.9
P22626	HNRNPA2B1	47	56381.1	1300.8	43.3	24	21786.4	60.8	358.0
P51991	HNRNPA3	33	53511.9	1252.9	42.7	19	20259.5	61.7	328.1
P07910	HNRNPC	26	51302.5	1212.5	42.3	31	98860.7	398.6	248.0
P31943	HNRNPH1	21	12736.5	718.6	17.7	20	19754.3	502.9	39.3
P61978	HNRNPK	45	17996.1	3184.3	5.7	40	37238.7	1191.4	31.3
P14866	HNRNPL	48	16274.2	804.4	20.2	44	21567.4	146.4	147.3
P52272	HNRNPM	24	3624.6	352.3	10.3	29	5806.1	35.0	166.1
O43390	HNRNPR	37	16275.7	463.9	35.1	33	24710.1	77.9	317.2
Q00839	HNRNPU	103	50213.0	1884.5	26.6	28	11012.5	53.9	204.5
Q1KMD3	HNRNPUL2	26	2392.4	441.1	5.4	24	4044.5	88.2	45.8
P26599	PTBP1	20	3160.9	641.8	4.9	33	10262.7	70.4	145.8
P38159	RBMX	7	40144.0	5631.9	7.1	18	37167.3	304.1	122.2
P62318	SNRPD3	5	6789.1	1659.4	4.1	4	2467.4	79.6	31.0
O60506	SYNCRIP	24	4843.1	137.1	35.3	26	6207.0	50.6	122.6
**Splicing factor**									
Q15424	SAFB	11	6169.1	769.2	8.0	8	3146.4	73.6	42.7
O75494	SRSF10	8	7494.1	836.0	9.0	7	8042.8	31.4	256.0
P84103	SRSF3	3	3325.3	818.3	4.1	4	3630.5	40.6	89.5
Q16629	SRSF7	5	3744.0	846.5	4.4	4	4127.3	23.9	172.9
P62995	TRA2B	11	6638.2	1129.5	5.9	10	16863.9	14.2	1188.9
**Ribosormal proteins**									
P62241	RPS8	9	3592.5	1124.1	3.2	7	2126.1	119.9	17.7
**Other RNA binding proteins**									
Q15717	ELAVL1	16	5595.7	661.7	8.5	9	6187.4	51.6	119.9
Q12905	ILF2	24	7513.3	457.9	16.4	31	12309.0	57.4	214.6
Q12906	ILF3	41	11003.5	894.7	12.3	40	20821.8	44.1	471.9
Q07666	KHDRBS1	14	11612.9	1495.6	7.8	10	37656.6	189.5	198.7
P43243	MATR3	34	8839.2	569.3	15.5	21	6177.7	29.5	209.8
Q9UKM9	RALY	15	13029.4	938.6	13.9	10	10403.6	41.7	249.6

Supplementary Table S1. List of APOBEC3B partner candidates identified in both AMO1 and RPMI8226 cells by LC-MS/MS immunoprecipitation. Unique proteins detected in mass spectrometric analysis of myeloma cell line immunoprecipitate. Only proteins with unique peptide count (95% confidence) ≧2, relative abundance ≧2000 and fold change ≧2 in both datasets are shown.

Supplementary Figure S5

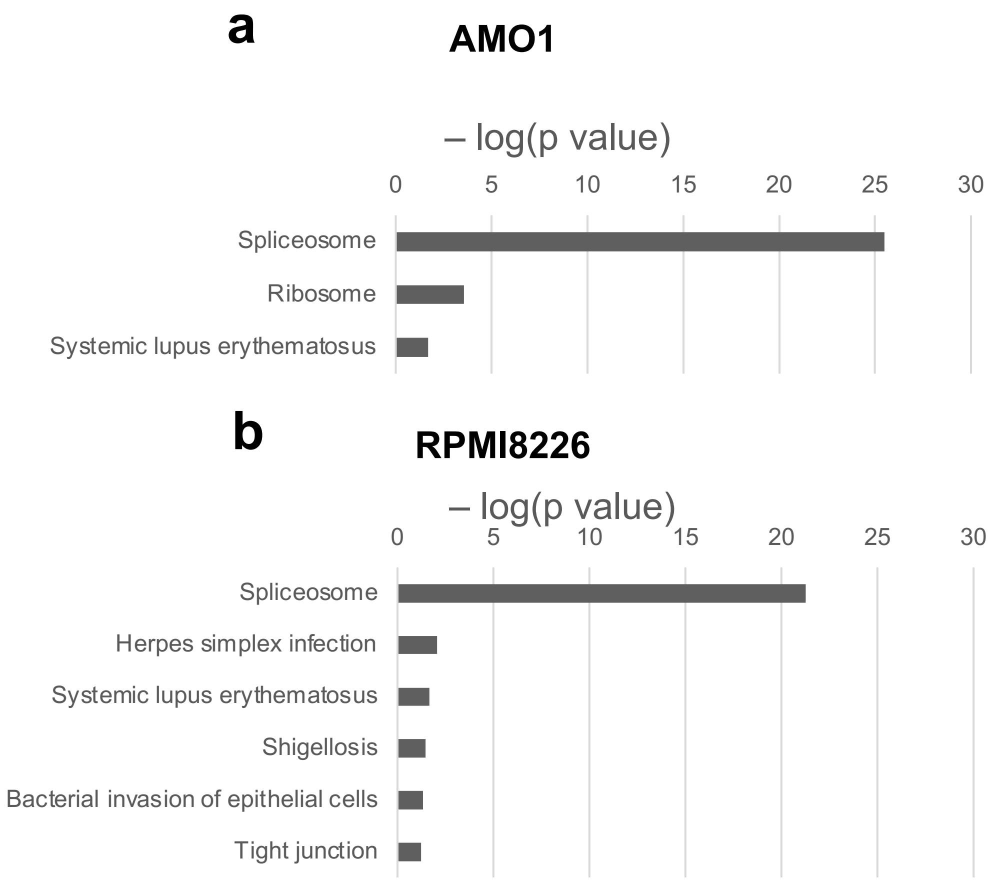

Supplementary Figure S5. Pathway analysis of putative A3B-interacting proteins. KEGG pathway analysis revealed that A3B-interacting protein candidates belong to the spliceosome and the ribosome. 55 interacting protein candidates identified in AMO1 (a) and 51 candidates in RPMI8226 (b) cells were analysed.

Supplementary Figure S6

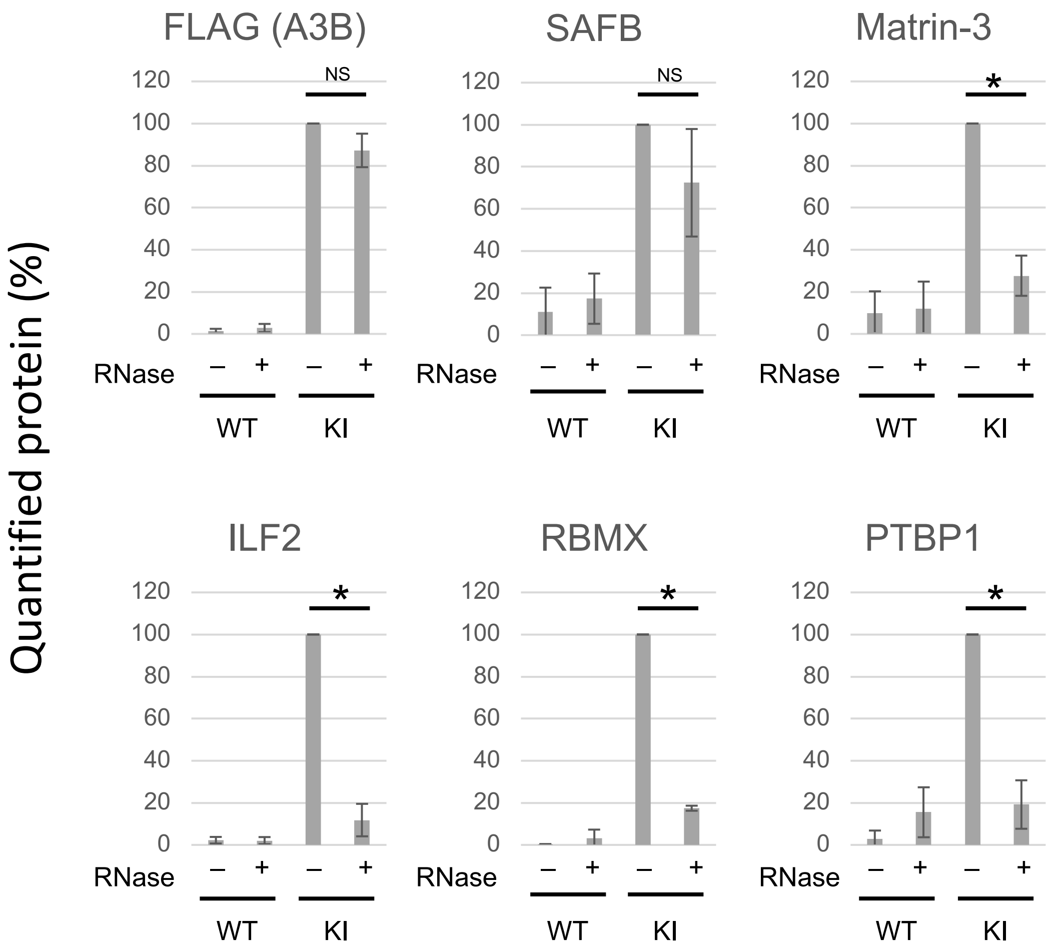

Supplementary Figure S6. Quantification of band intensity of FLAG-IP lysates from AMO1 cells. Quantification of immunoblot band intensity in three biologically independent experiments (including those shown in Figure 2) of representative interacting protein candidates in AMO1 cells. All the values are normalized by those of AMO1-KI without RNase. Values are means ± standard deviations (error bar). Asterisks (*) show statistically significant difference ($p < 0.05$).

Supplementary Figure S7

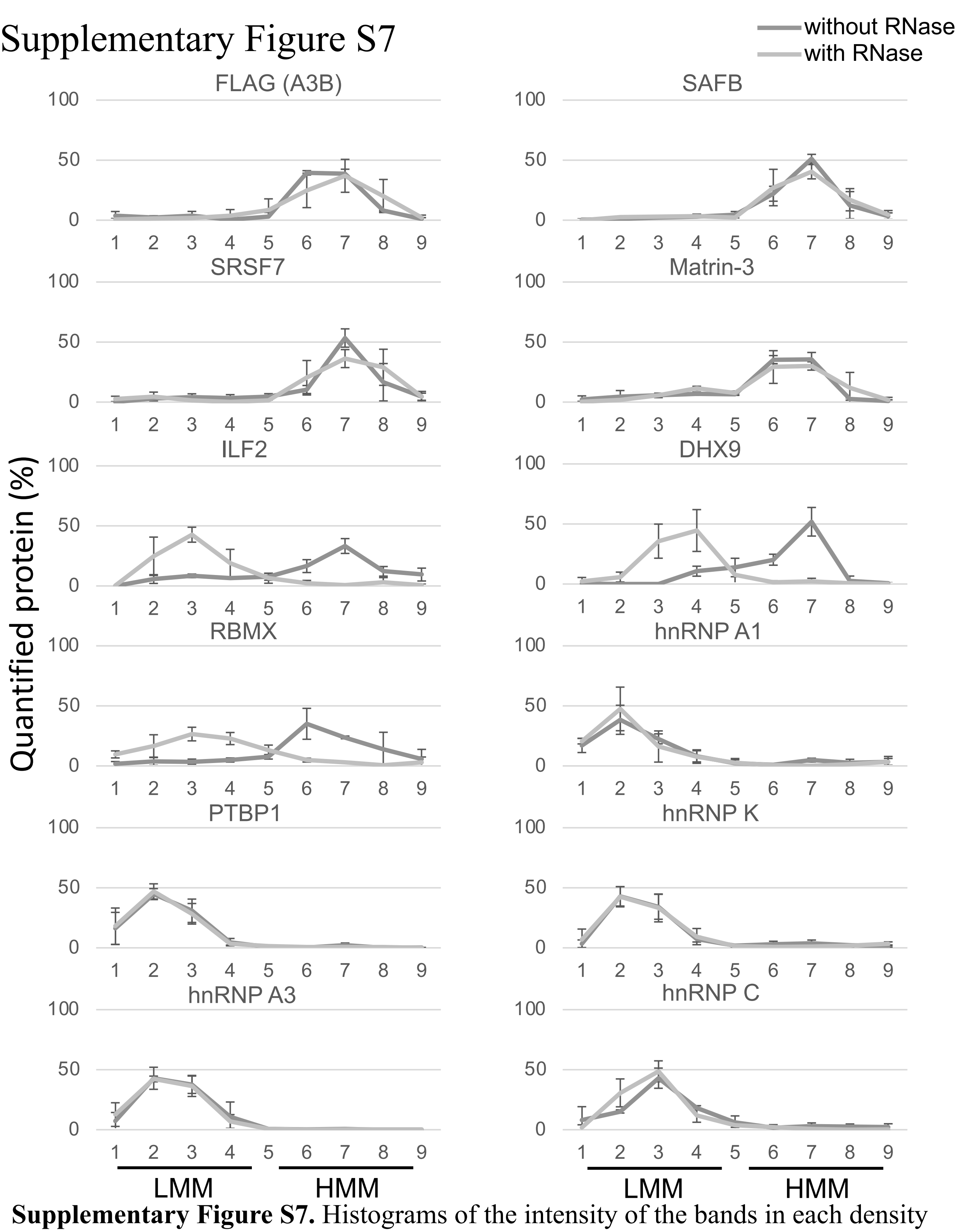

Supplementary Figure S7. Histograms of the intensity of the bands in each density gradient fraction from AMO1 cells, as shown in Figure 3. The band intensity of three biologically independent Western blot experiments was quantified (including those shown in Figure 3). Values are means ± standard deviations (error bar). *LMM*, low molecular mass. *HMM*, high molecular mass.

Supplementary Figure S8

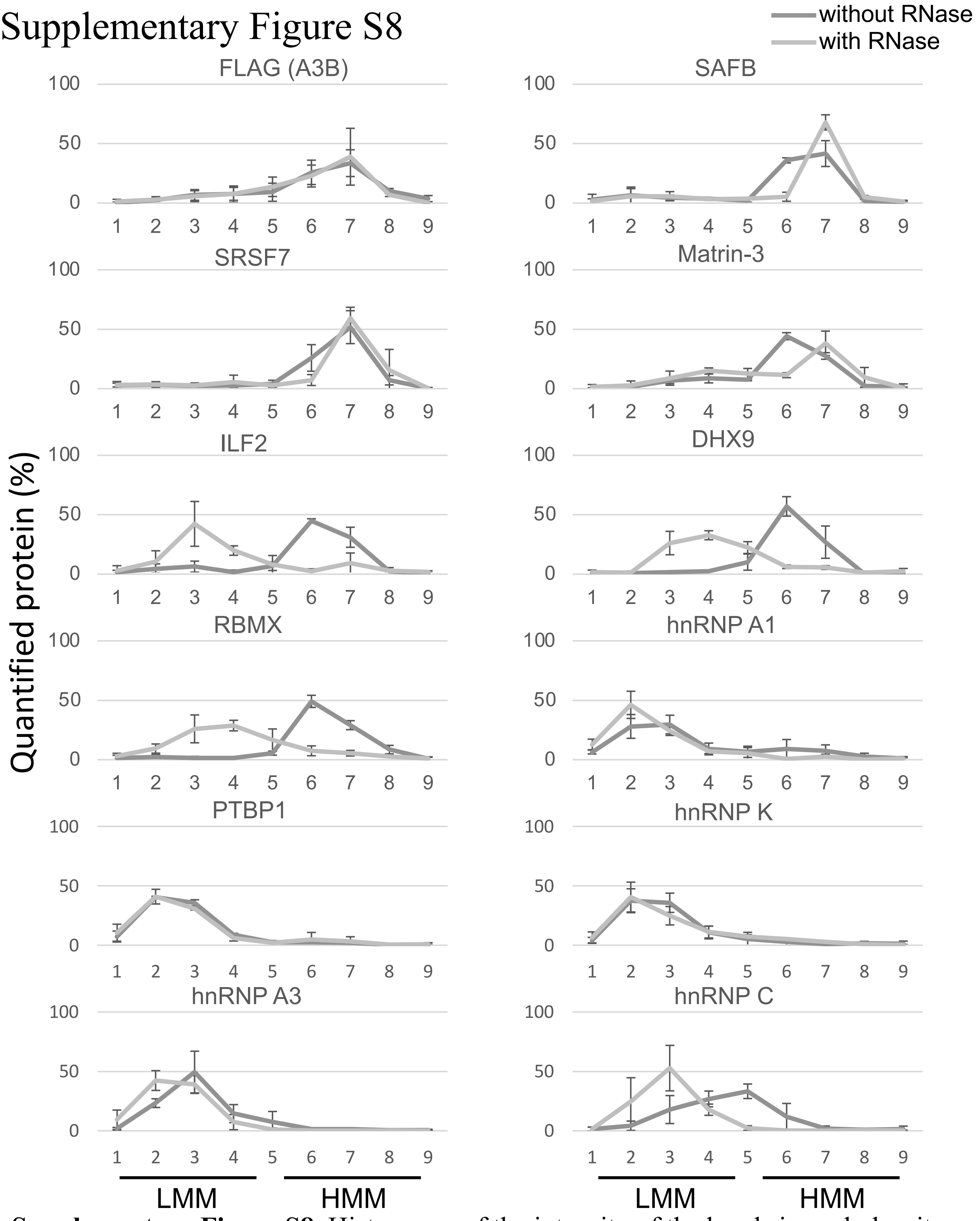

Supplementary Figure S8. Histograms of the intensity of the bands in each density gradient fraction from RPMI8226 cells, as shown in Figure 3. The band intensity of three biologically independent Western blot experiments was quantified (including those shown in Figure 3). Values are means ± standard deviations (error bar). *LMM*, low molecular mass. *HMM*, high molecular mass.

Supplementary Figure S9

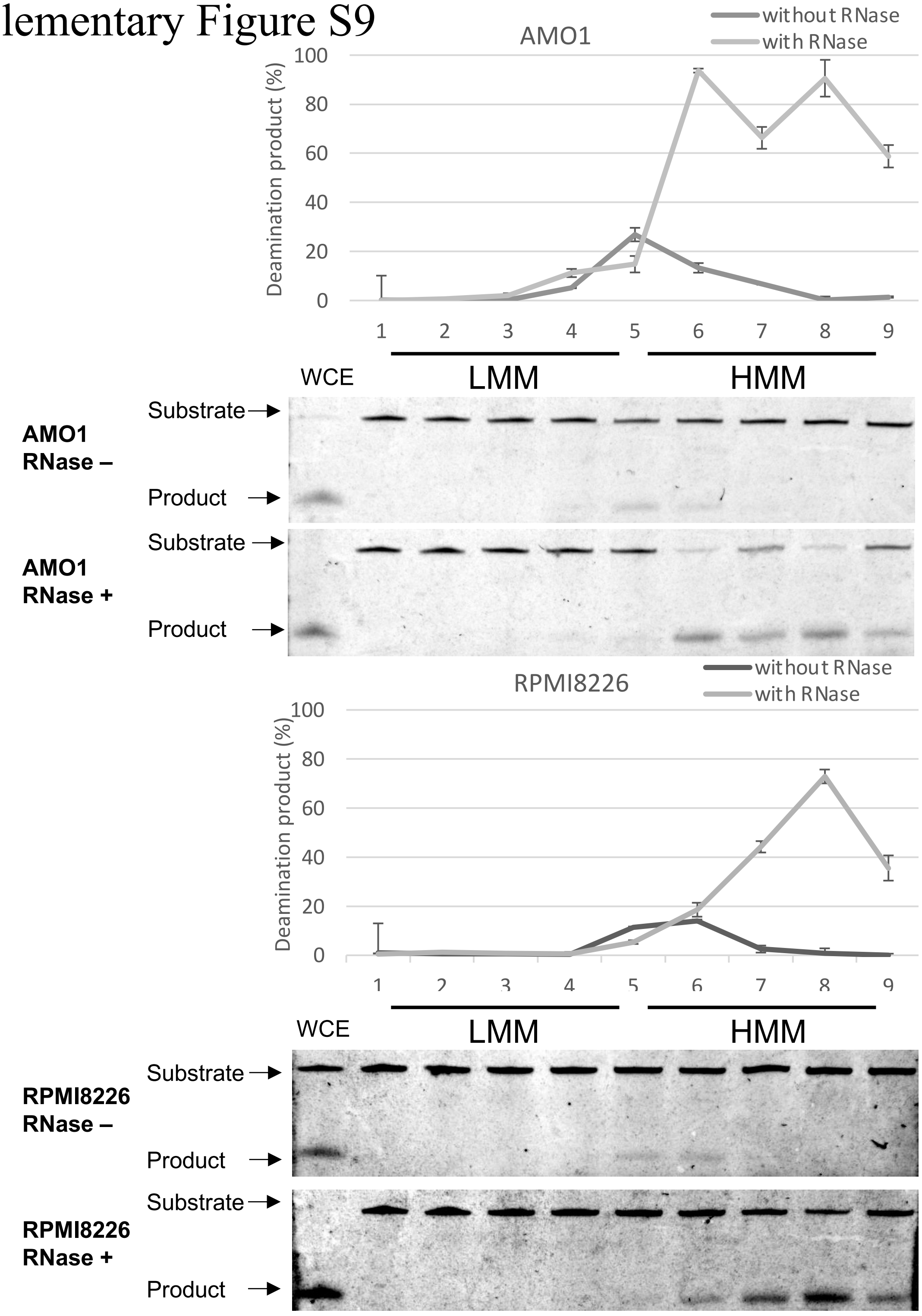

Supplementary Figure S9. RNase treatment enhances A3B cytidine deaminase activity in high-density fractions. The fractions obtained after density gradient sedimentation (Figure 3) were subjected to gel-based CDA assays. Deaminated/total (deaminated and aminated) percentage of product in three biologically independent experiments was calculated using the ImageJ software. Values are means ± standard deviations (error bar). *LMM*, low molecular mass. *HMM*, high molecular mass. Original gels are presented in Supplementary Figure S17.

Supplementary Figure S10

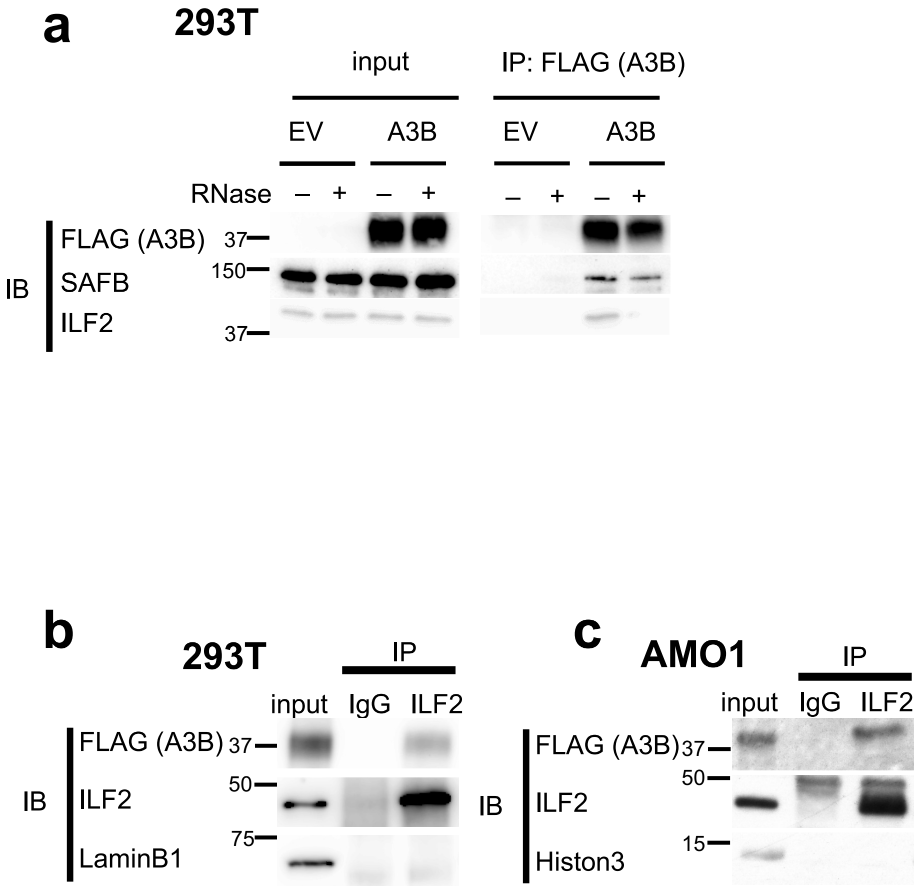

Supplementary Figure S10. Validation of the interaction with A3B by co-immunoprecipitation and reciprocal co-immunoprecipitation of nuclear extracts. (a, b) HEK293T cells were transfected with expression vectors for A3B-3×FLAG. Nuclear extracts from these cells were co-immunoprecipitated with anti-FLAG (a) or anti-ILF2 (b) antibodies, with or without RNase A, followed by immunoblotting with the indicated antibodies. (c) Nuclear extracts from AMO1-KI cells were co-immunoprecipitated with anti-ILF2 antibodies, with or without RNase A, followed by immunoblotting with the indicated antibodies. Images shown here are representative of three independent experiments. *EV,* empty vector. Original blots are presented in Supplementary Figure S18.

Supplementary Figure S11

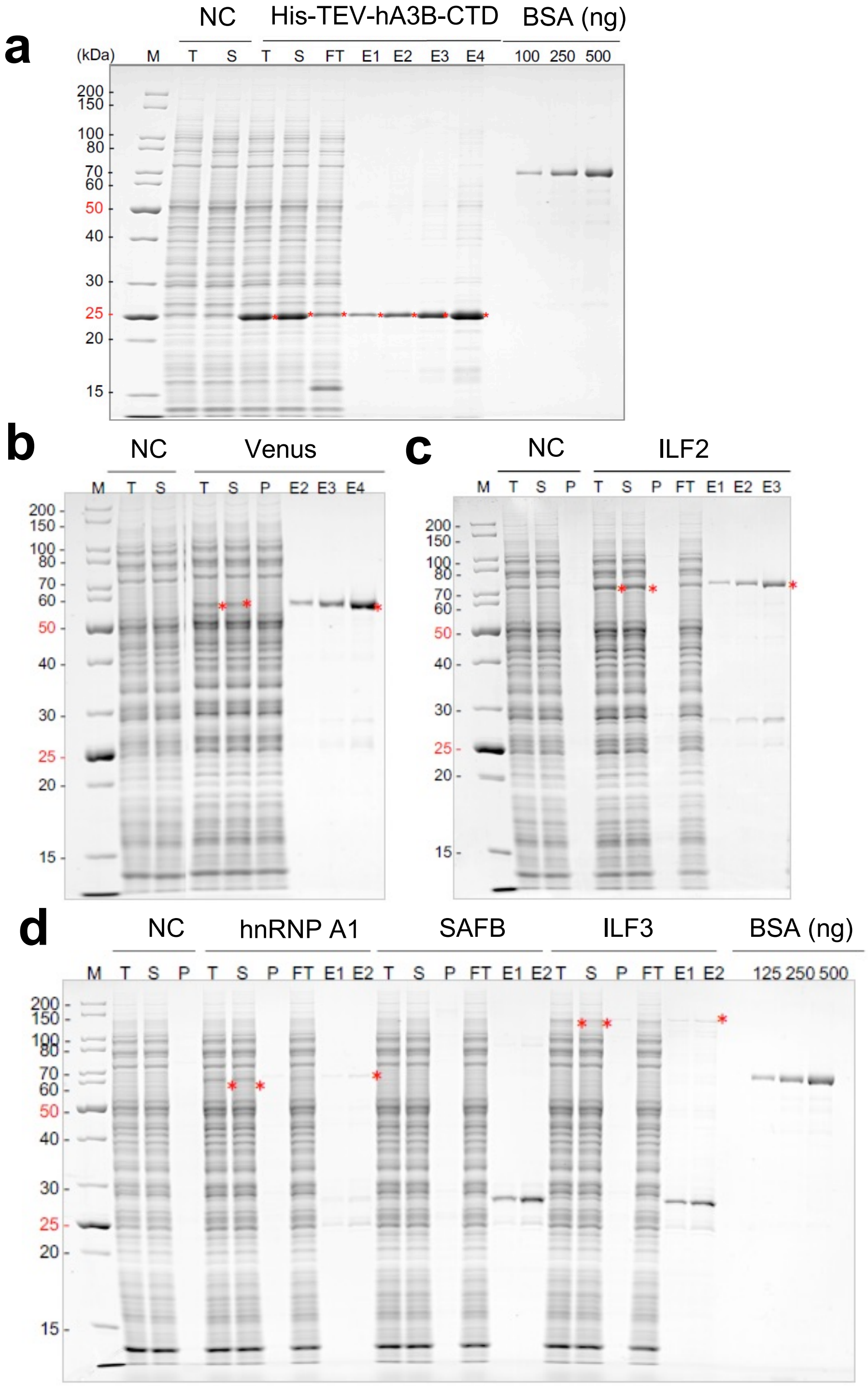

Supplementary Figure S11. Coomassie-stained gels illustrating the purity of A3B and ILF2 used in Supplementary Figure S12. SDS-PAGE followed by Coomassie staining was performed using purified (a) A3B, (b) Venus, (c) ILF2, (d) hnRNP A1, SAFB and ILF3. SAFB could not be purified. M, molecular weight marker. *NC*, negative control. *T*, total fraction. *S,* Supernatant fraction after centrifugation of the total fraction. *P*, Precipitation fraction after centrifugation of the total fraction. *FT*, Flow Through fraction. *E*, Eluent (*E1*, 0.625 µL. *E2*, 1.25 µL. *E3*, 2.5 µL, *E4*, 5 µL). * Detected protein.

Supplementary Figure S12

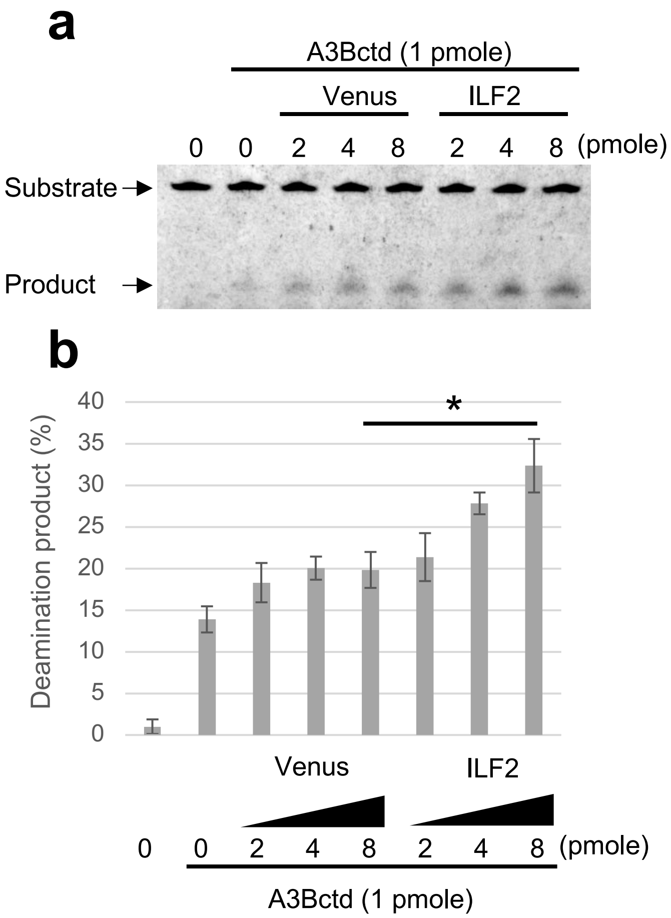

Supplementary Figure S12. A3B cytidine deaminase activity was enhanced by purified ILF2. (a) Gel-based CDA assay using purified proteins. Representative TBE-urea PAGE result is shown. (b) Quantification of the cytidine deaminase activity data in three biologically independent experiments. Values are means ± standard deviations (error bar). Original gel is presented in Supplementary Figure S19.

Supplementary Figure S13-1
Original blots for Figure 2 (AMO1)

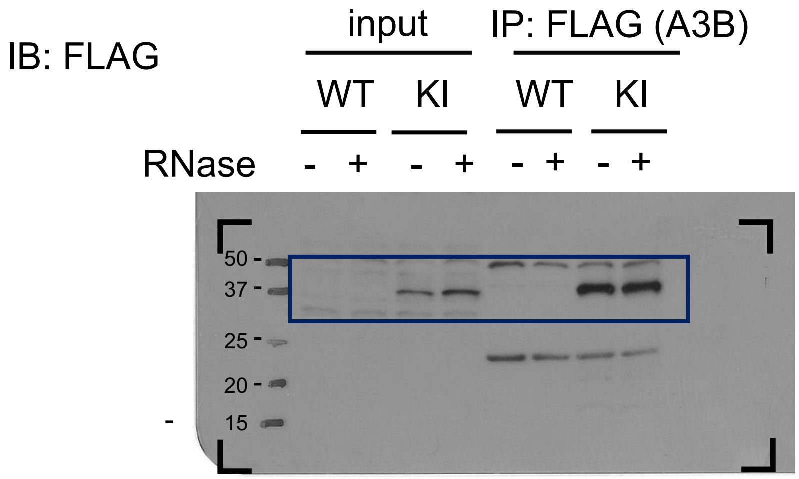

* cut prior to hybridisation with the antibody

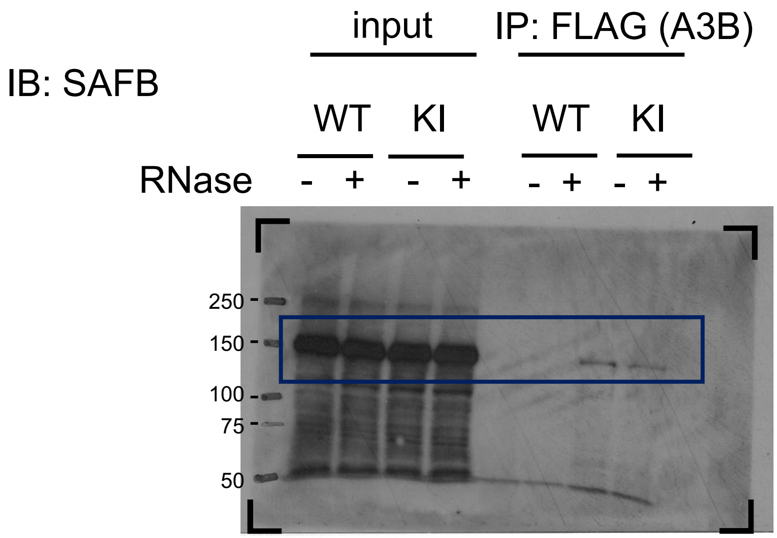

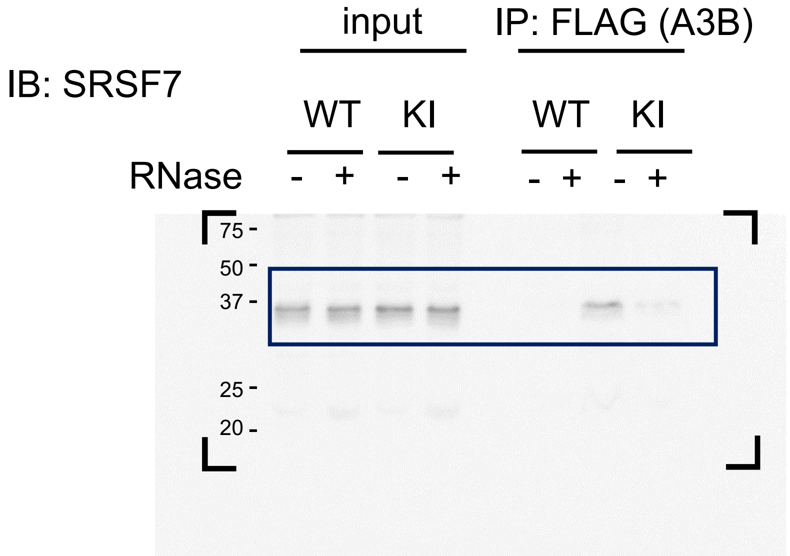

* cut prior to hybridisation with the antibody

Supplementary Figure S13-2
Original blots for Figure 2 (AMO1)

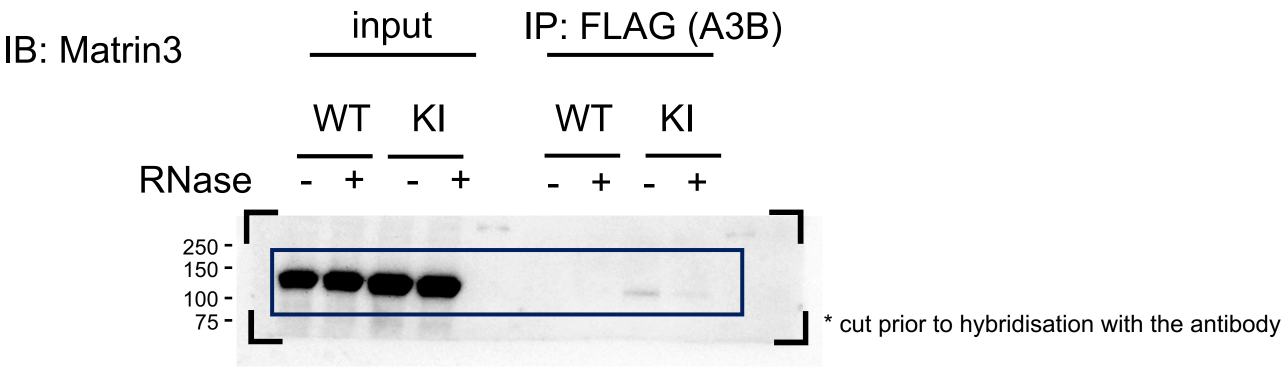

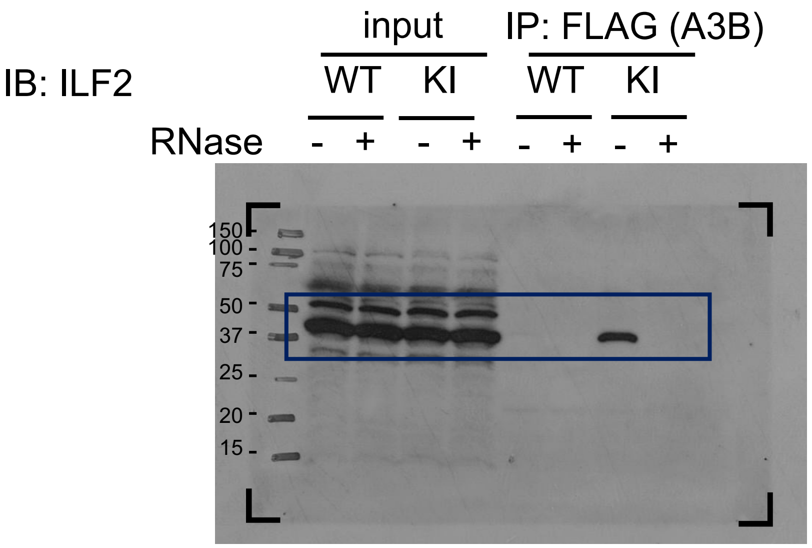

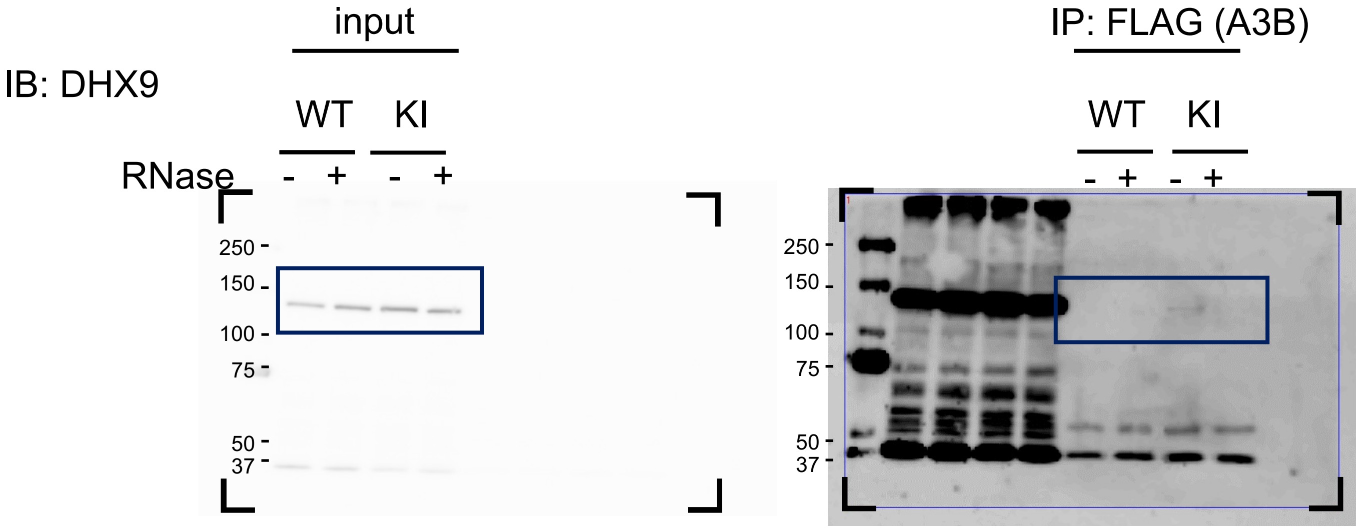

(short exposure) (long exposure)

Supplementary Figure S13-3
Original blots for Figure 2 (AMO1)

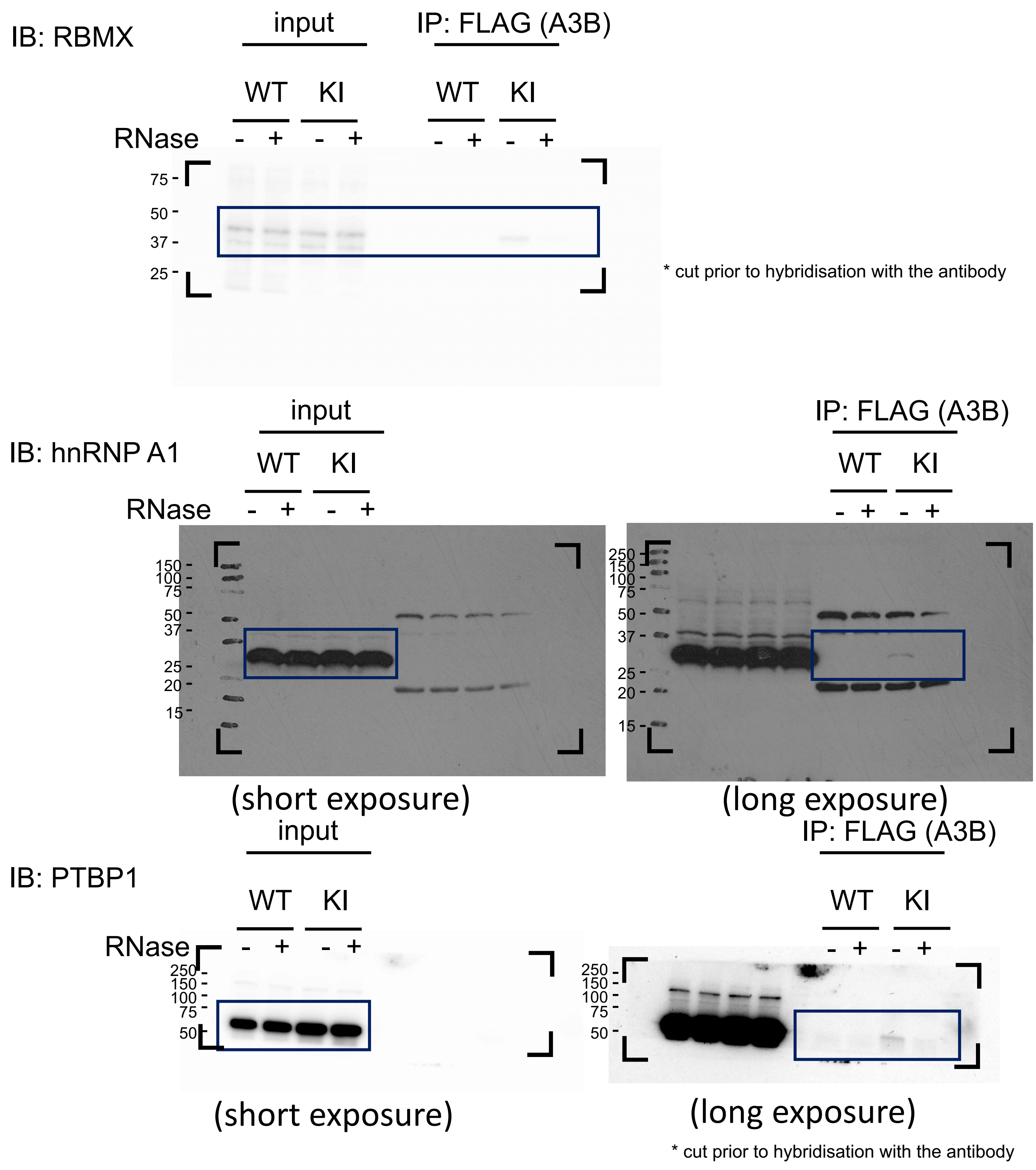

Supplementary Figure S13-4
Original blots for Figure 2 (AMO1)

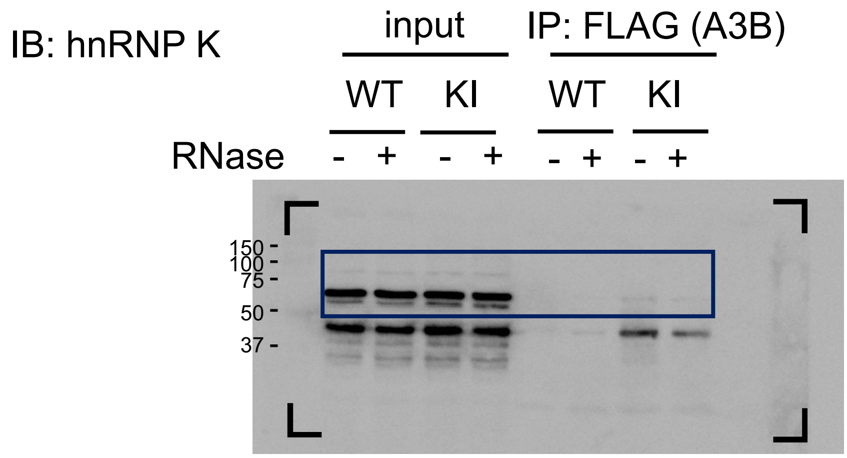

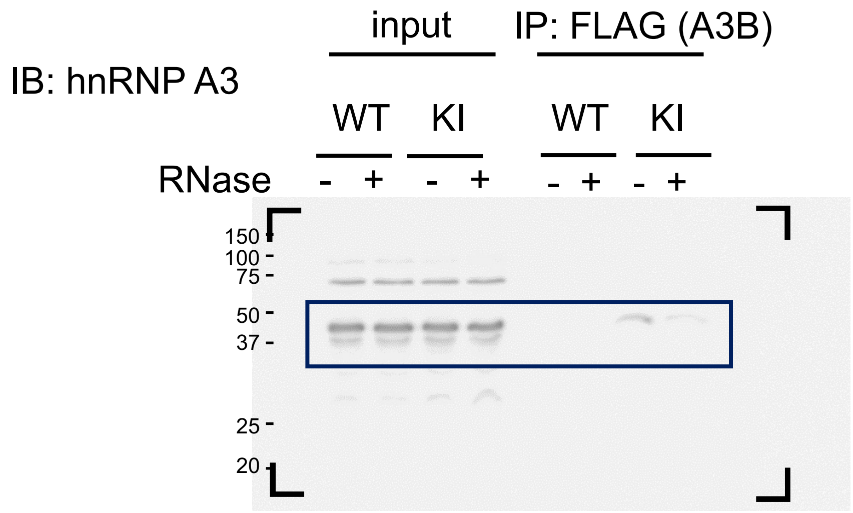

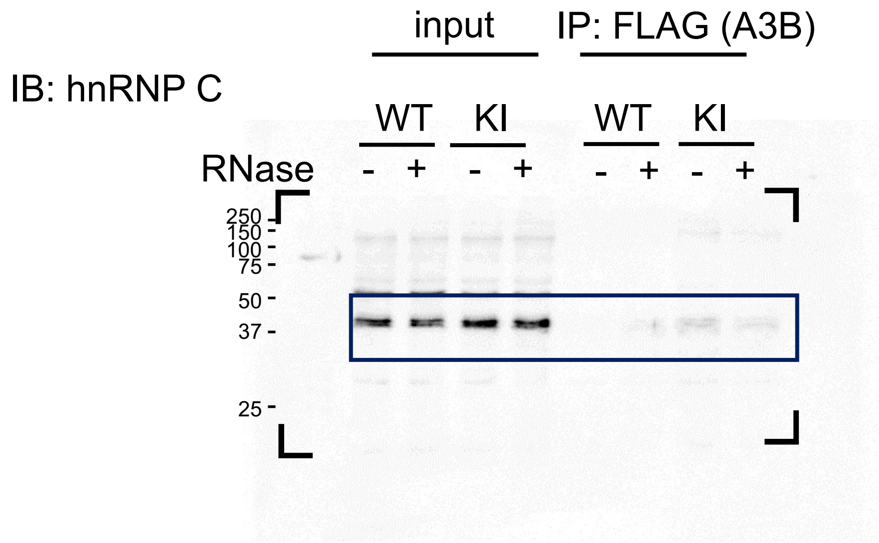

Supplementary Figure S13-5
Original blots for Figure 2 (RPMI8226)

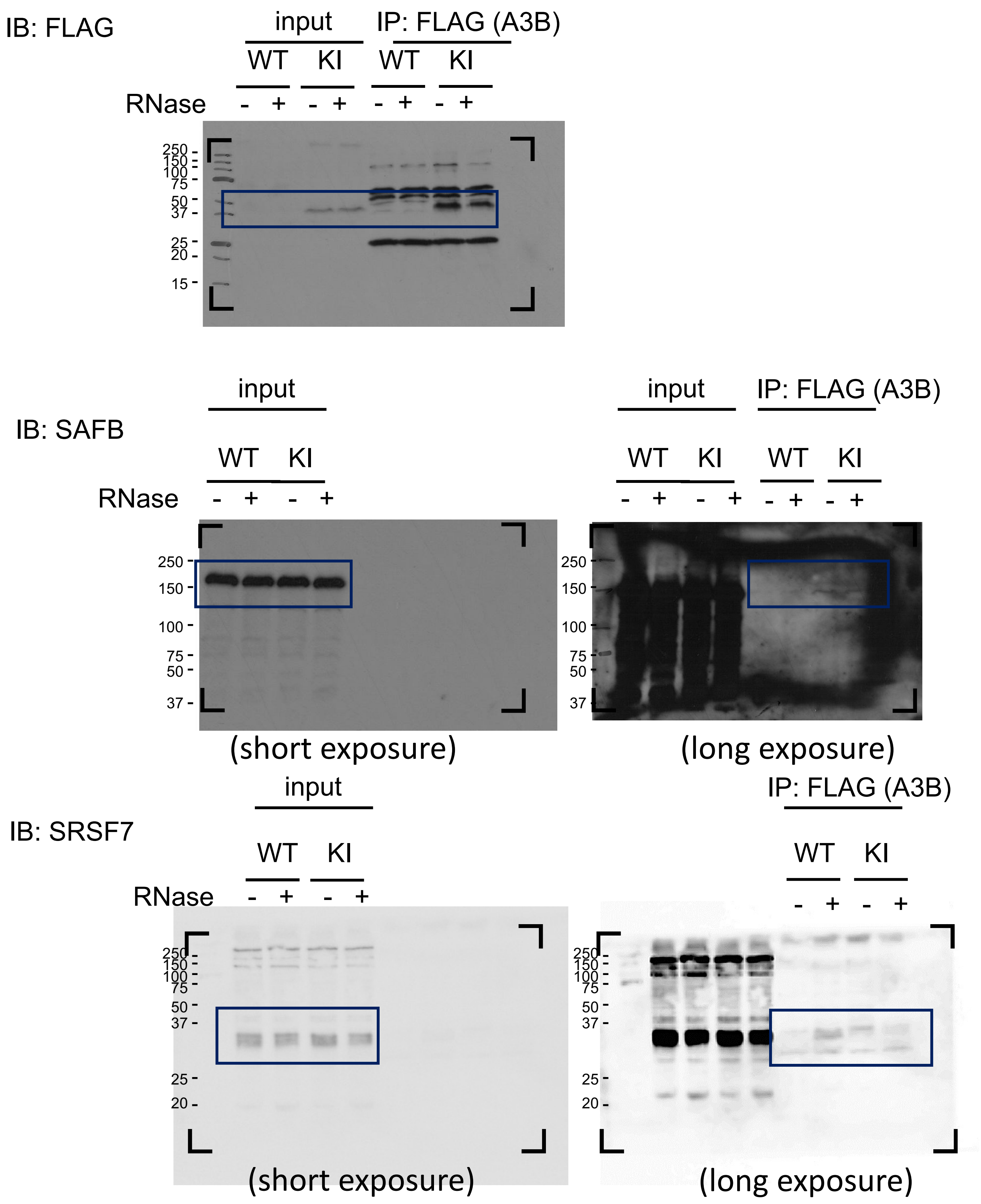

Supplementary Figure S13-6
Original blots for Figure 2 (RPMI8226)

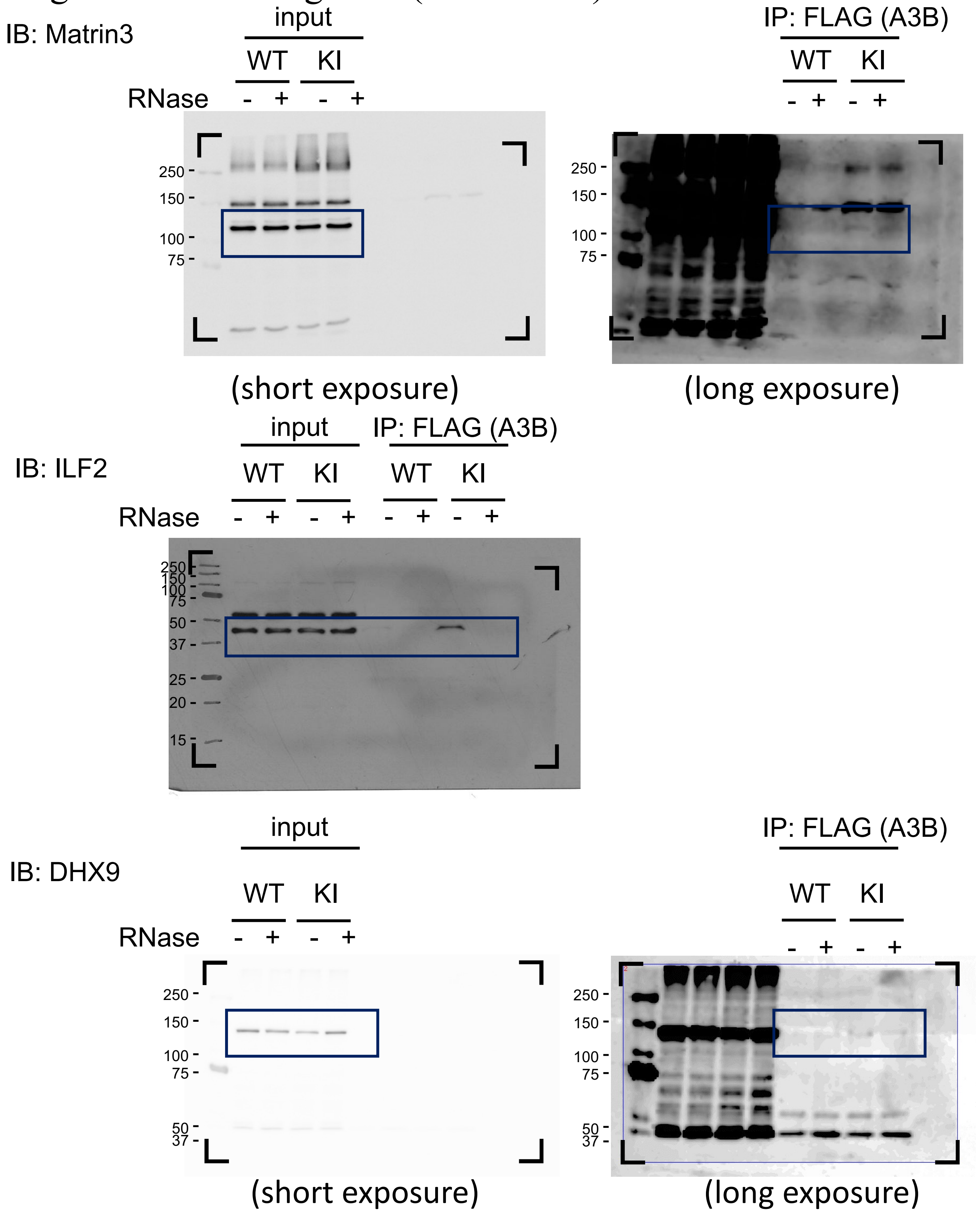

Supplementary Figure S13-7
Original blots for Figure 2 (RPMI8226)

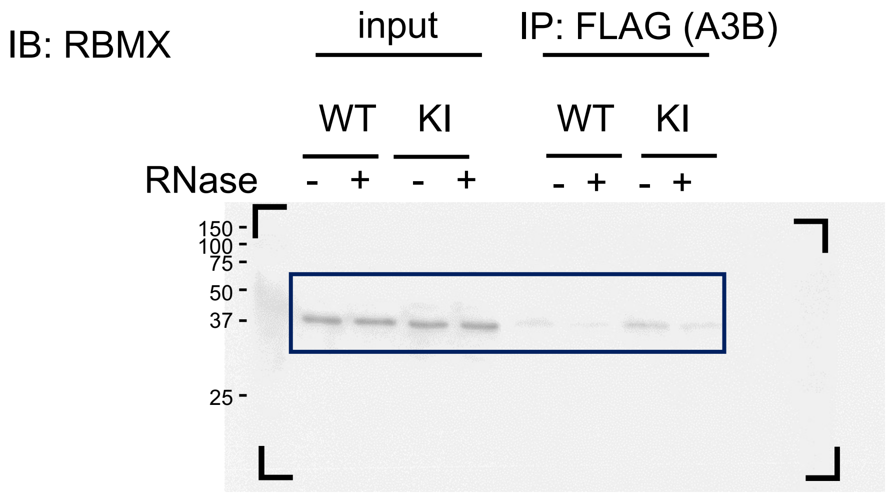

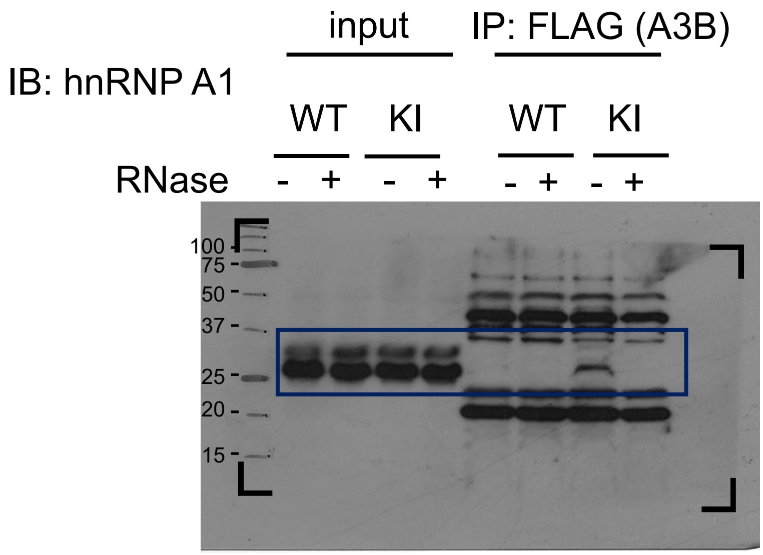

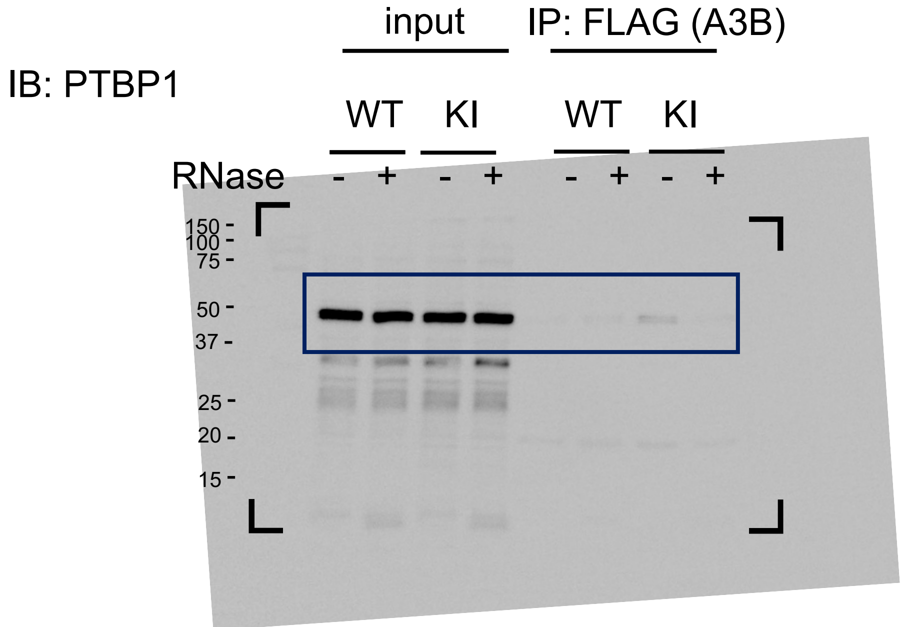

Supplementary Figure S13-8
Original blots for Figure 2 (RPMI8226)

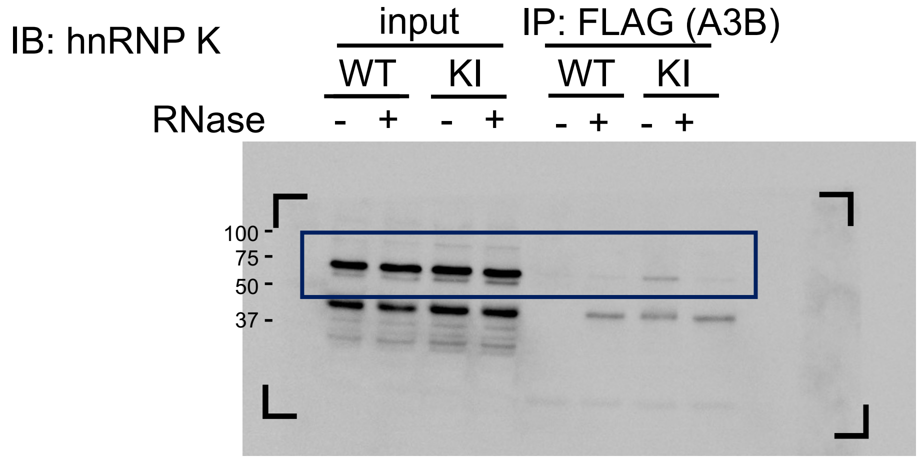

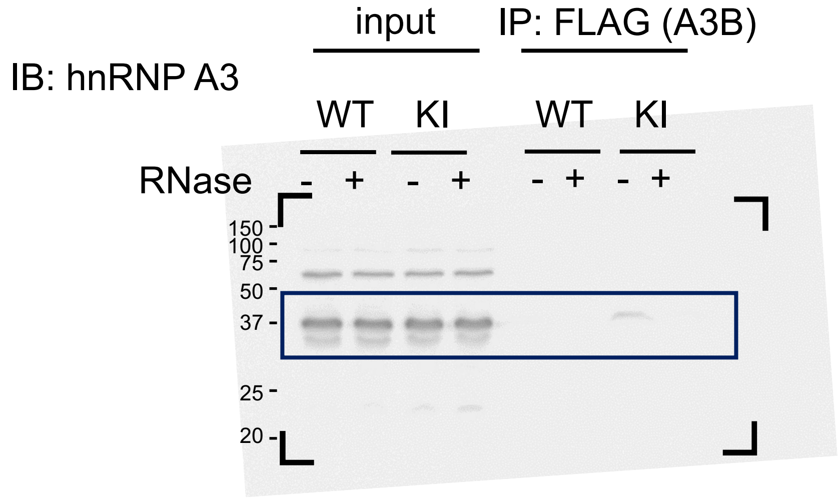

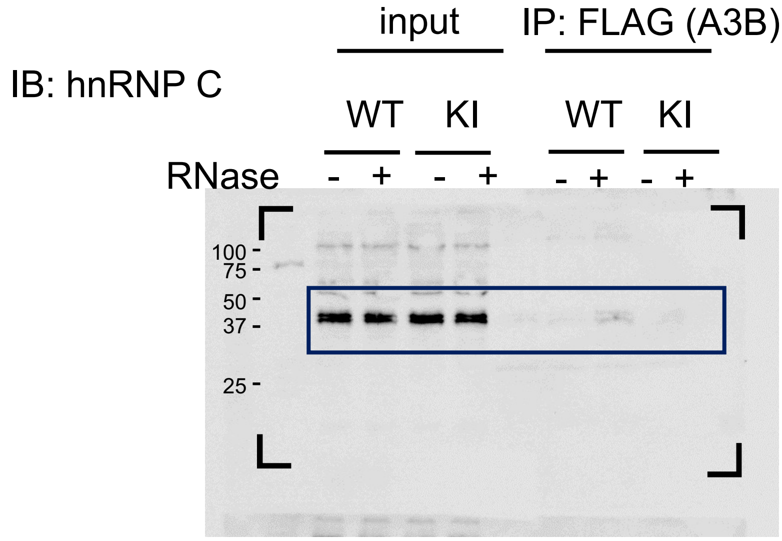

Supplementary Figure S14-1
Original blots for Figure 3 (AMO1)

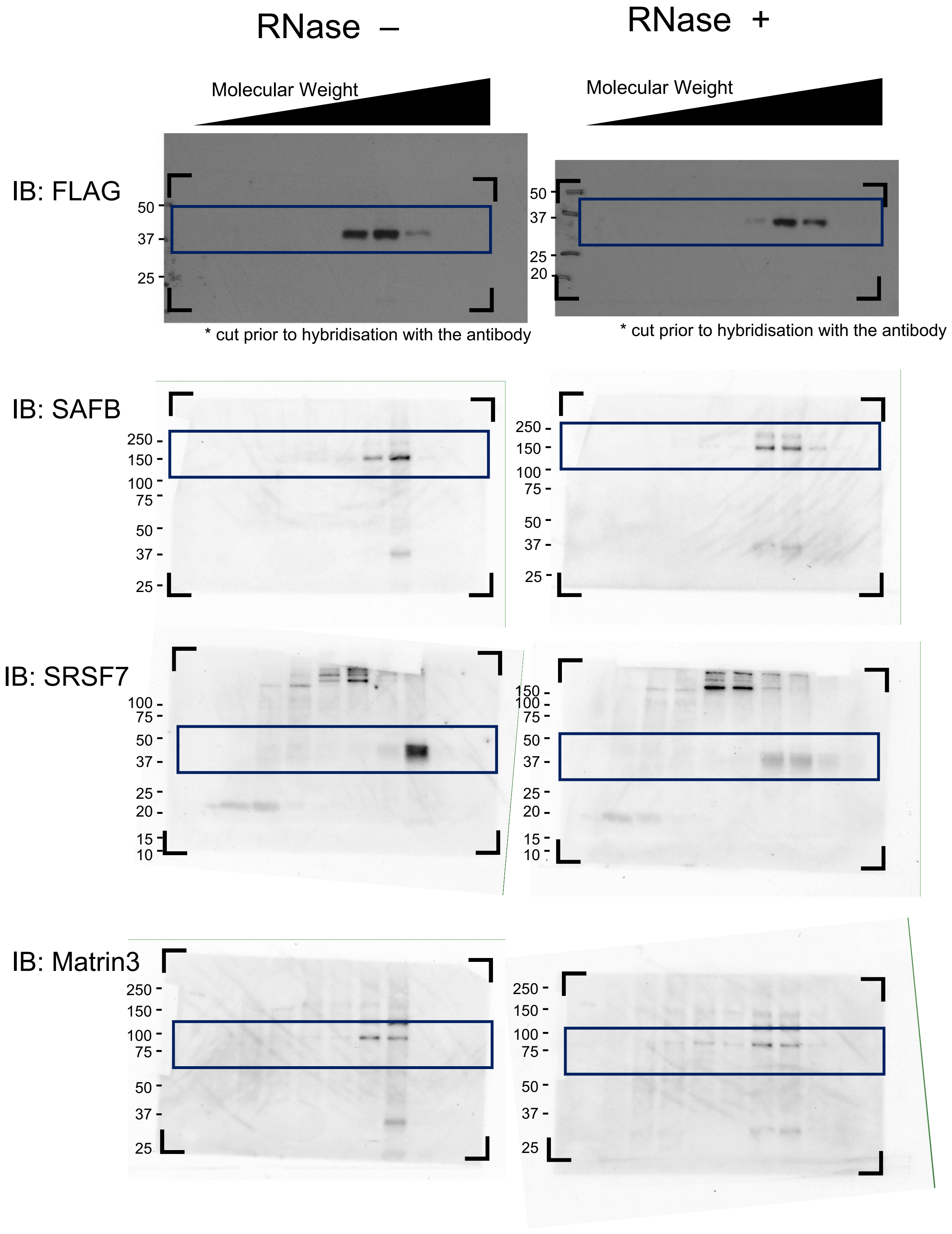

Supplementary Figure S14-2
Original blots for Figure 3 (AMO1)

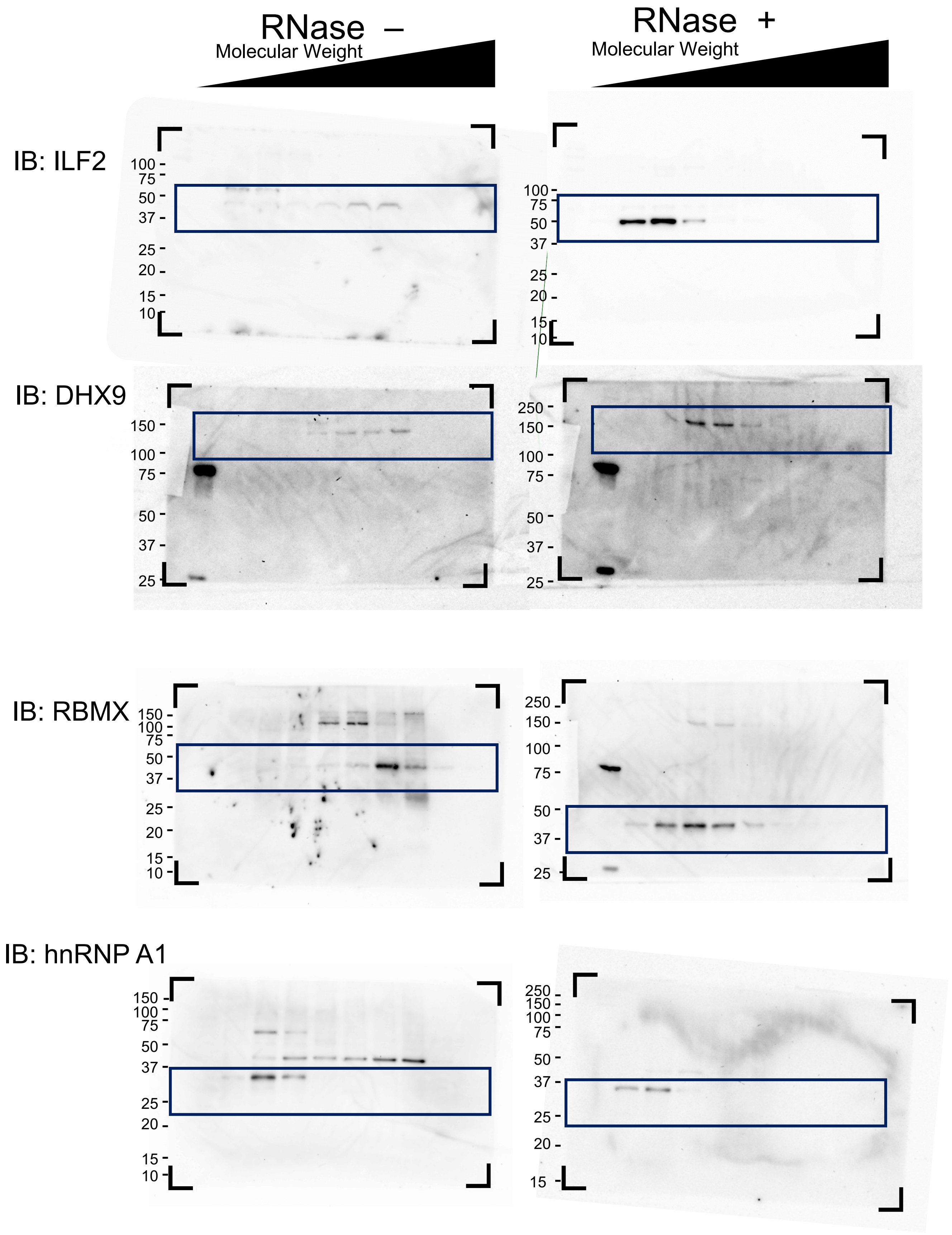

Supplementary Figure S14-3
Original blots for Figure 3 (AMO1)

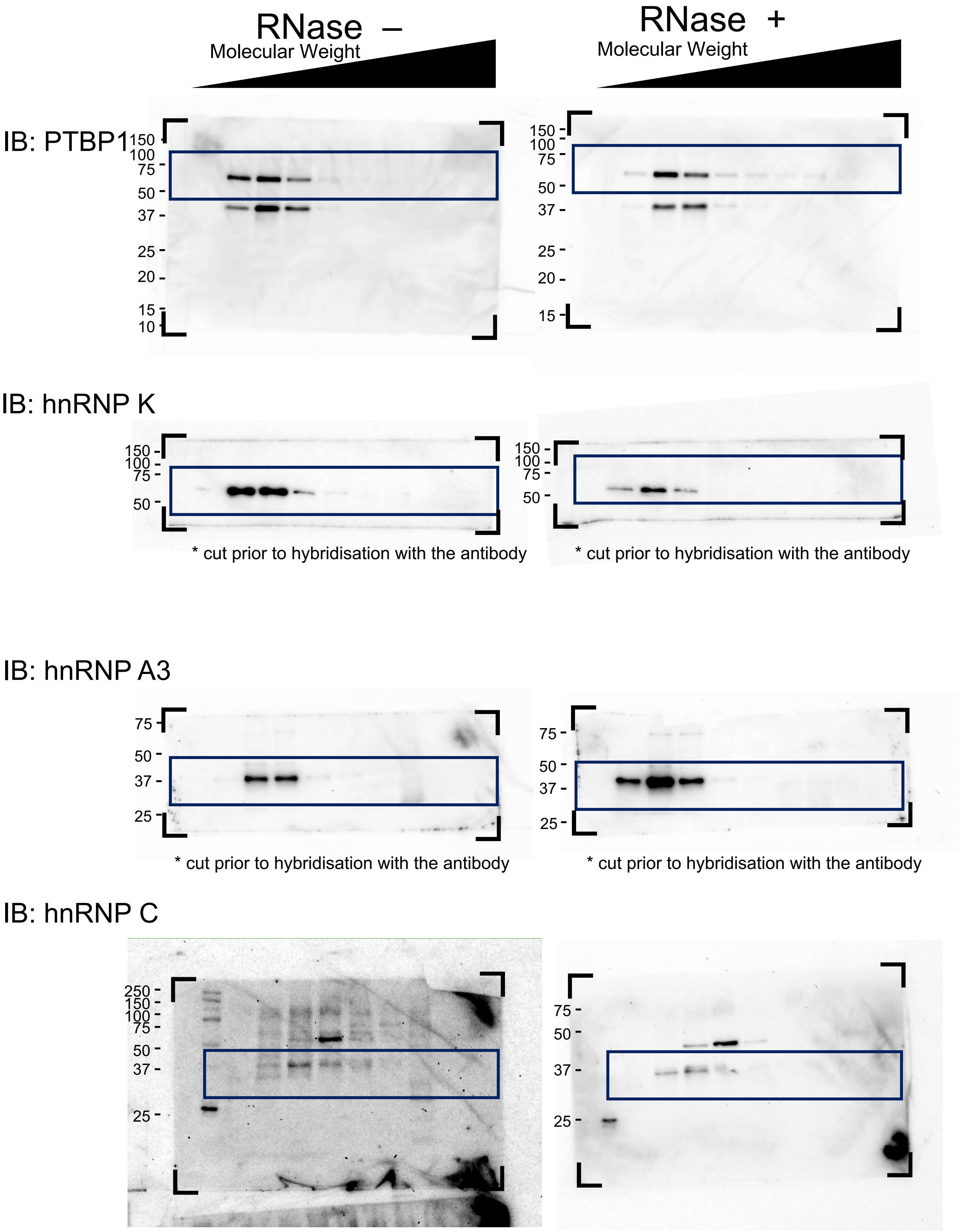

Supplementary Figure S14-4
Original blots for Figure 3 (RPMI8226)

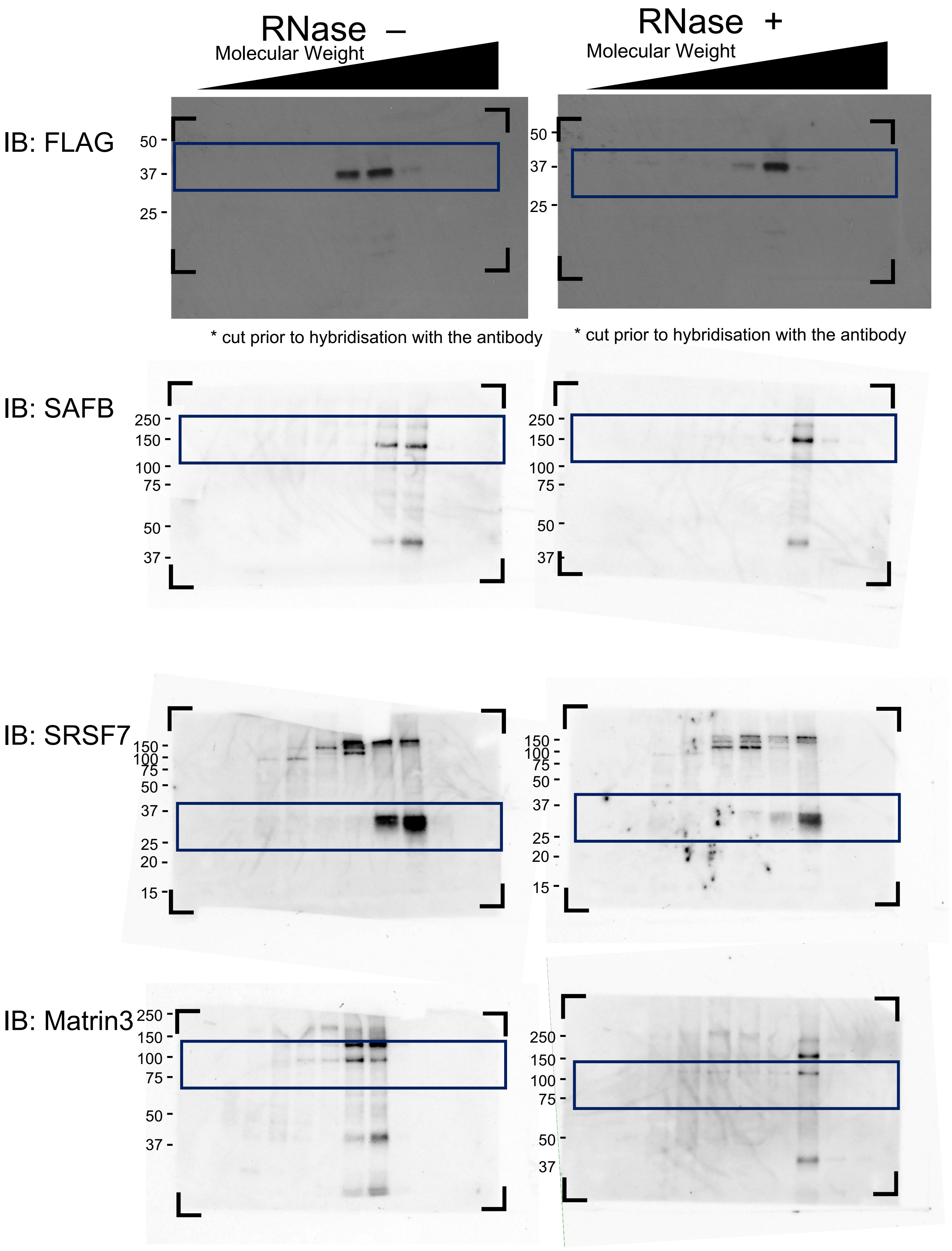

Supplementary Figure S14-5
Original blots for Figure 3 (RPMI8226)

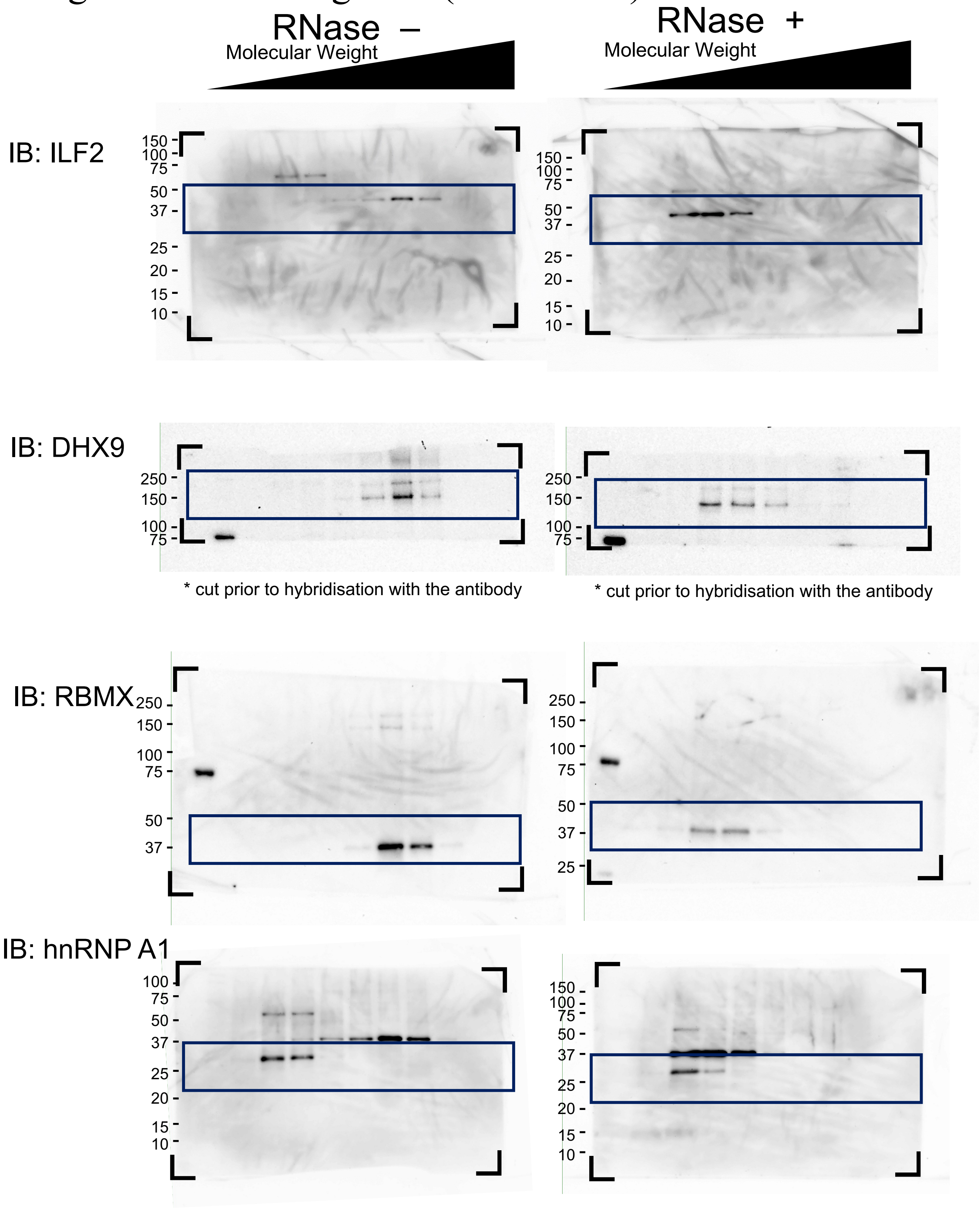

Supplementary Figure S14-6
Original blots for Figure 3 (RPMI8226)

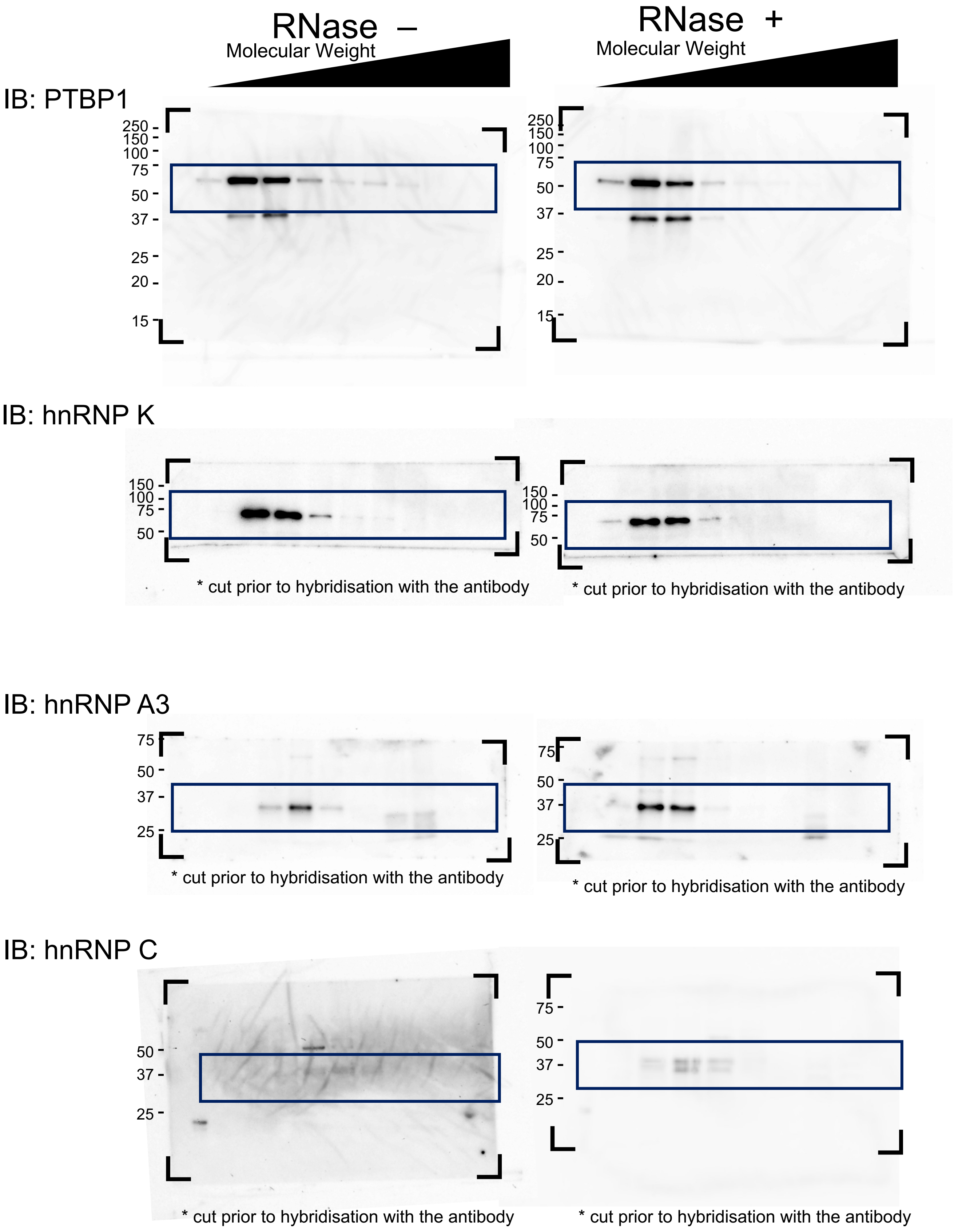

Supplementary Figure S14-7
Original silver-stained gel for Figure 3 (ALB)

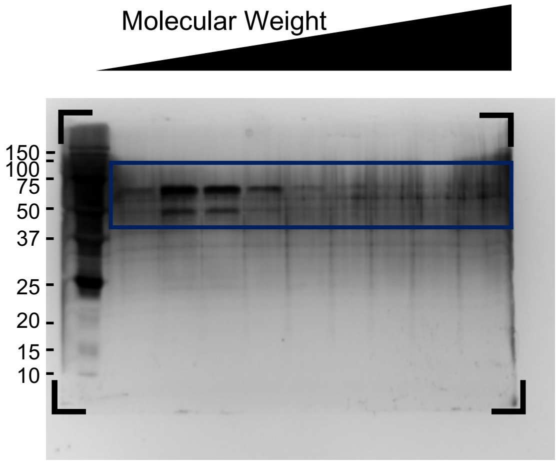

Supplementary Figure S15-1
Original blots and gel for Figure 5a

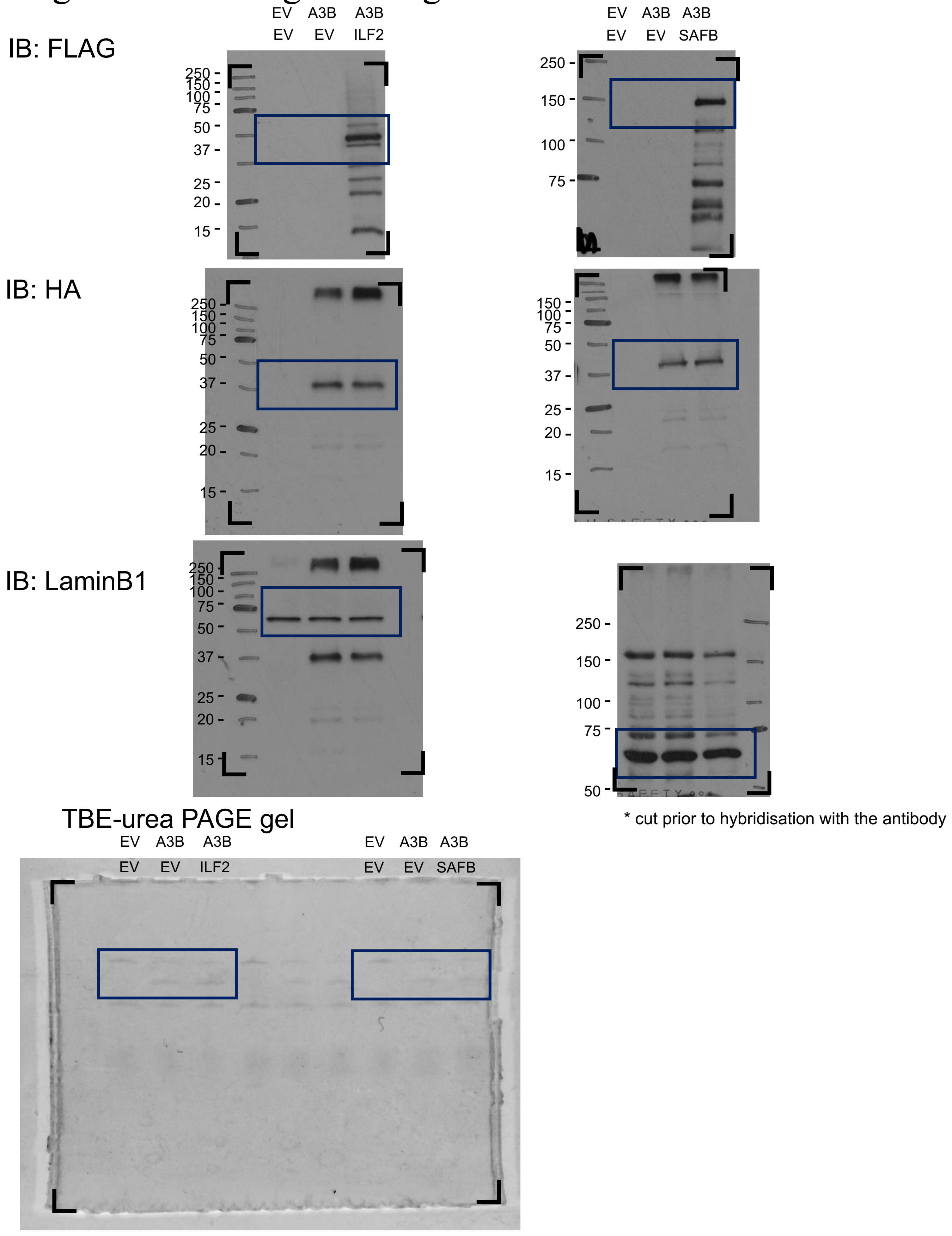

Supplementary Figure S15-2
Original blots and gel for Figure 5c

IB: FLAG

EV A3B A3B
NC NC si ILF2

EV A3B A3B
NC NC si SAFB

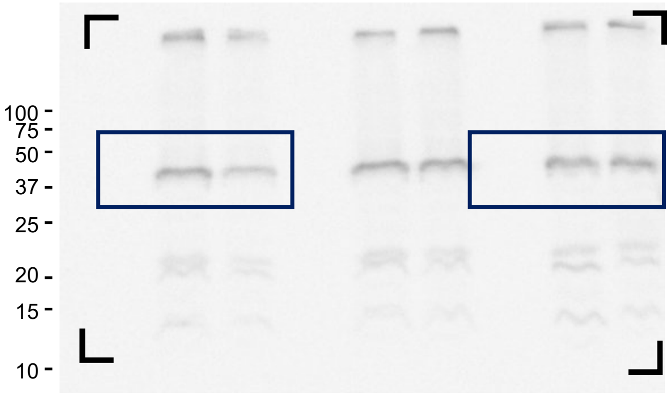

IB: LaminB1

EV A3B A3B
NC NC si ILF2

EV A3B A3B
NC NC si SAFB

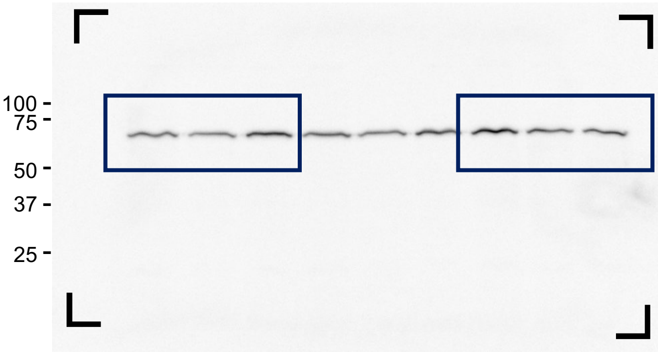

IB: ILF2

EV A3B A3B
NC NC si ILF2

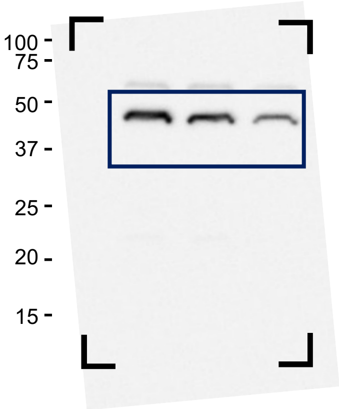

IB: SAFB

EV A3B A3B
NC NC si SAFB

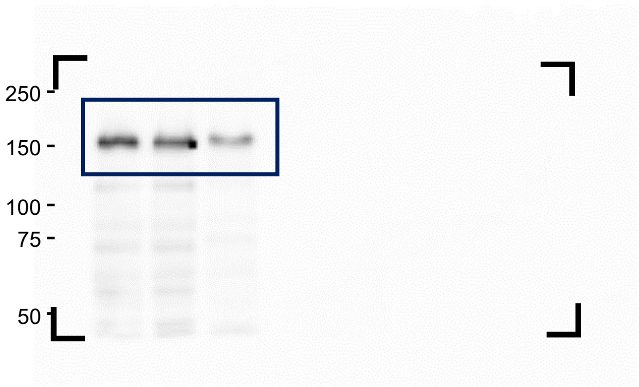

TBE-urea PAGE gel

EV A3B A3B
NC NC si ILF2

EV A3B A3B
NC NC si SAFB

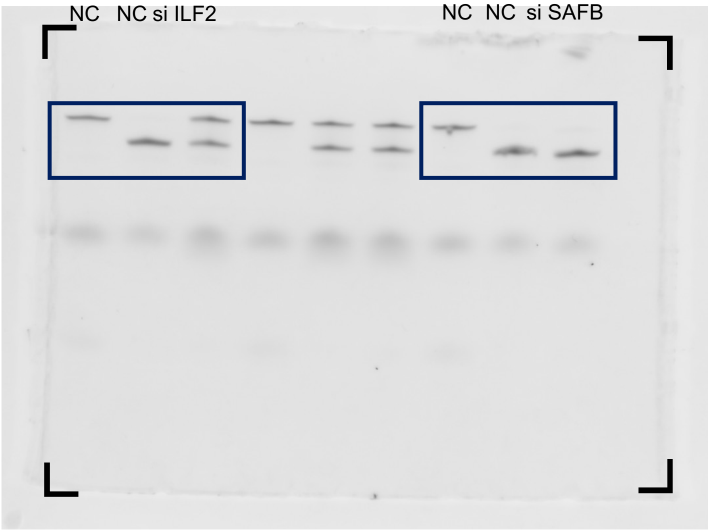

Supplementary Figure S16-1
Original blots and gel for Supplementary Figure S1a

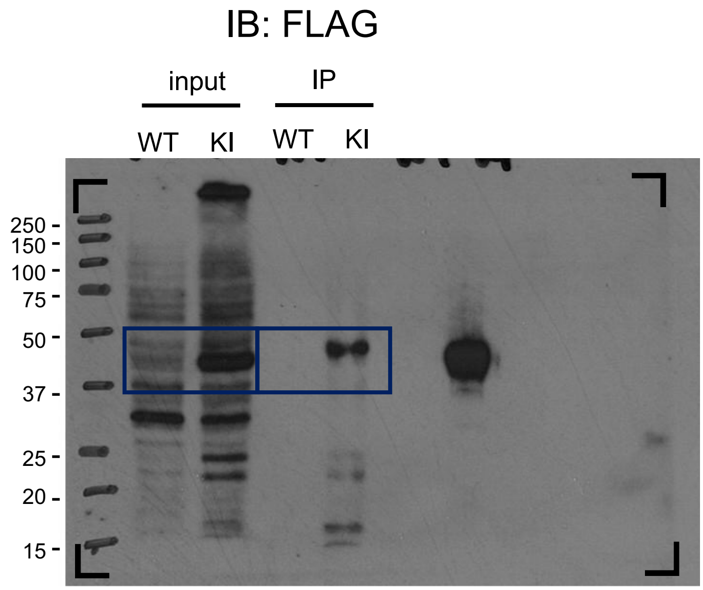

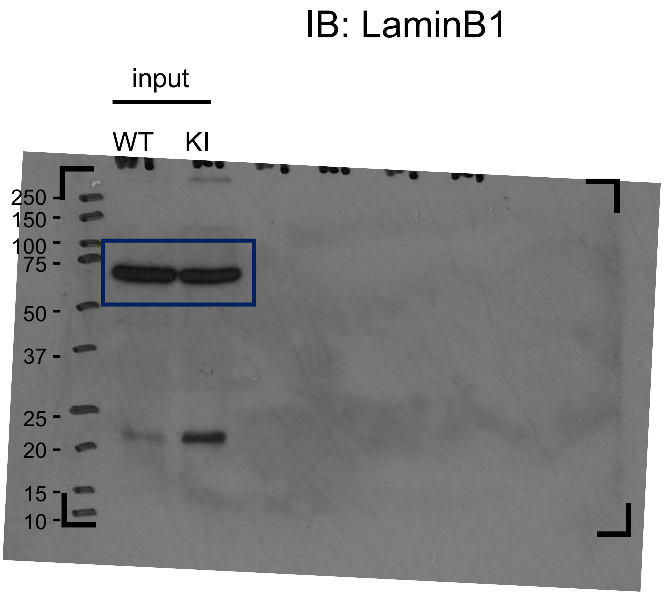

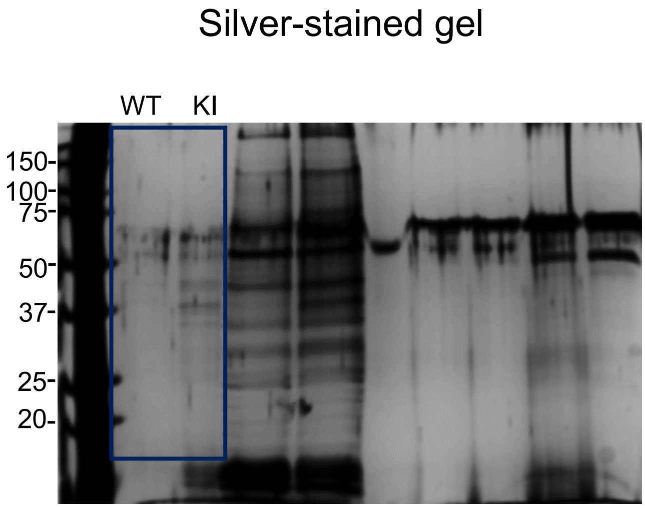

Supplementary Figure S16-2
Original blots and gel for Supplementary Figure S1b

IB: FLAG

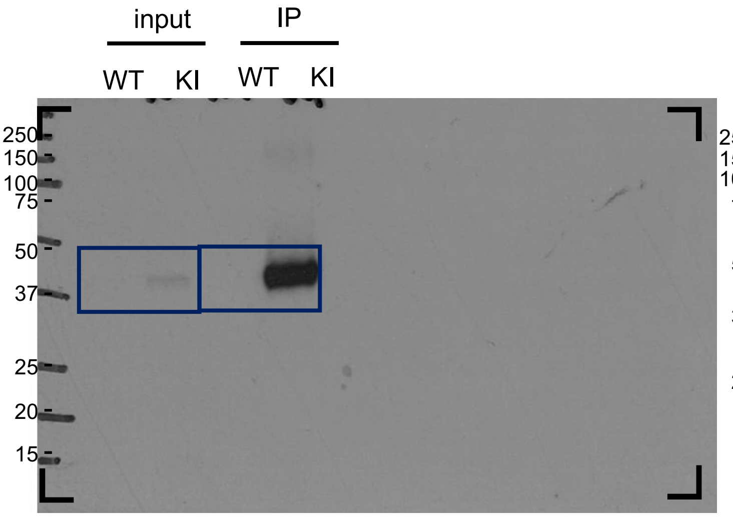

IB: LaminB1

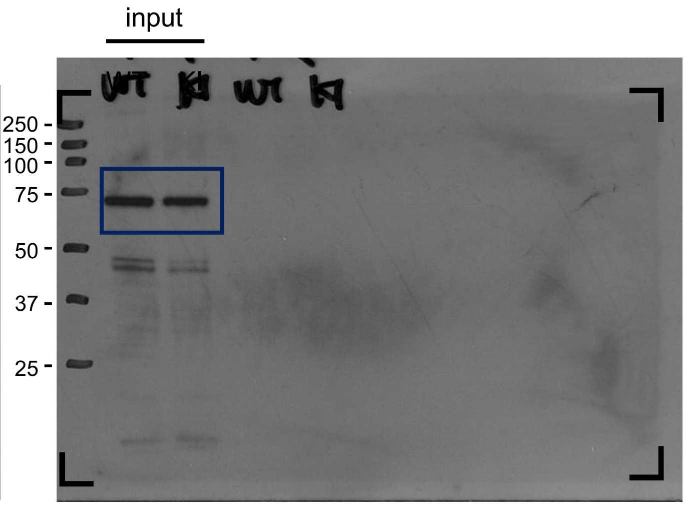

Silver-stained gel

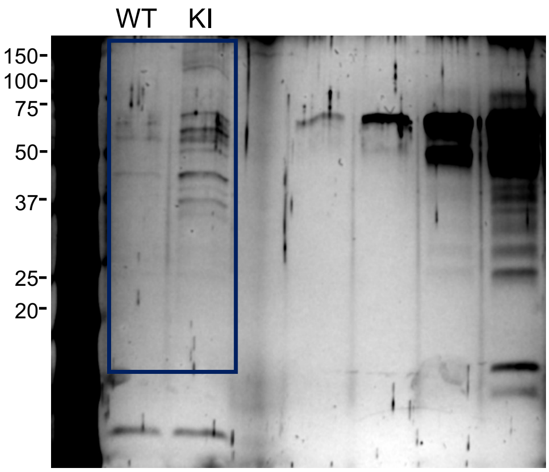

Supplementary Figure S17

Original gels for Supplementary Figure S9

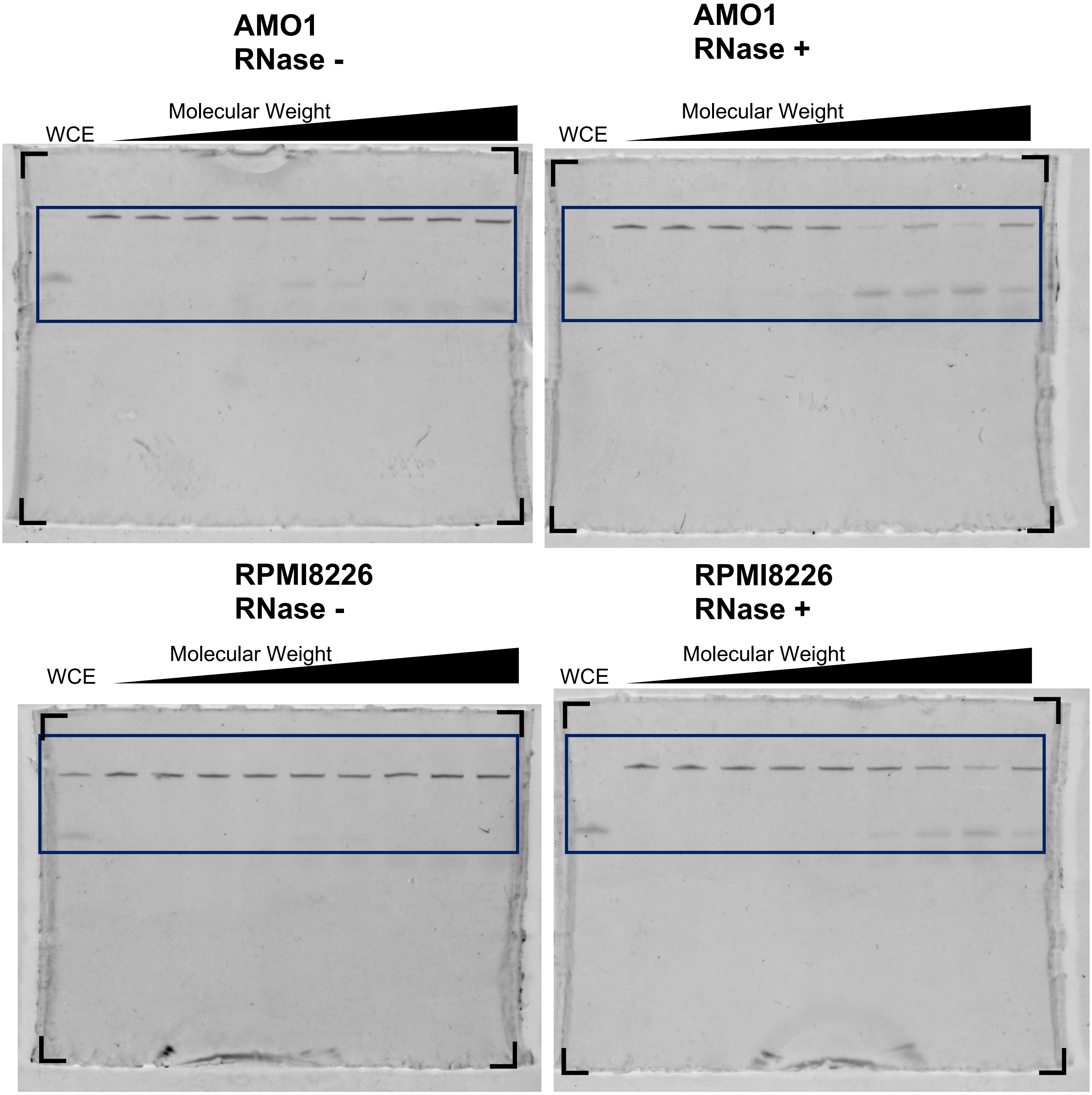

Supplementary Figure S18-1
Original blots for Supplementary Figure S10a

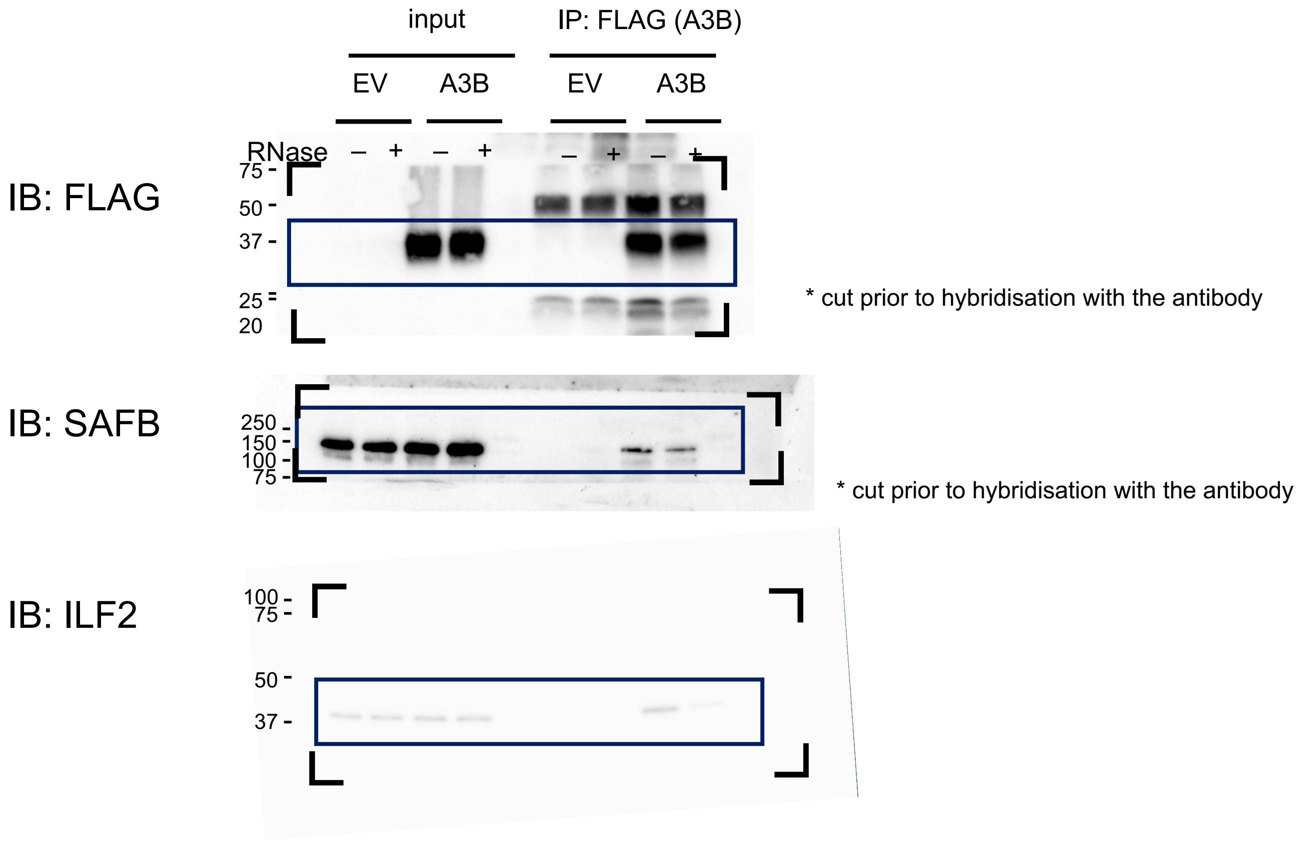

Supplementary Figure S18-2
Original blots for Supplementary Figure S10b

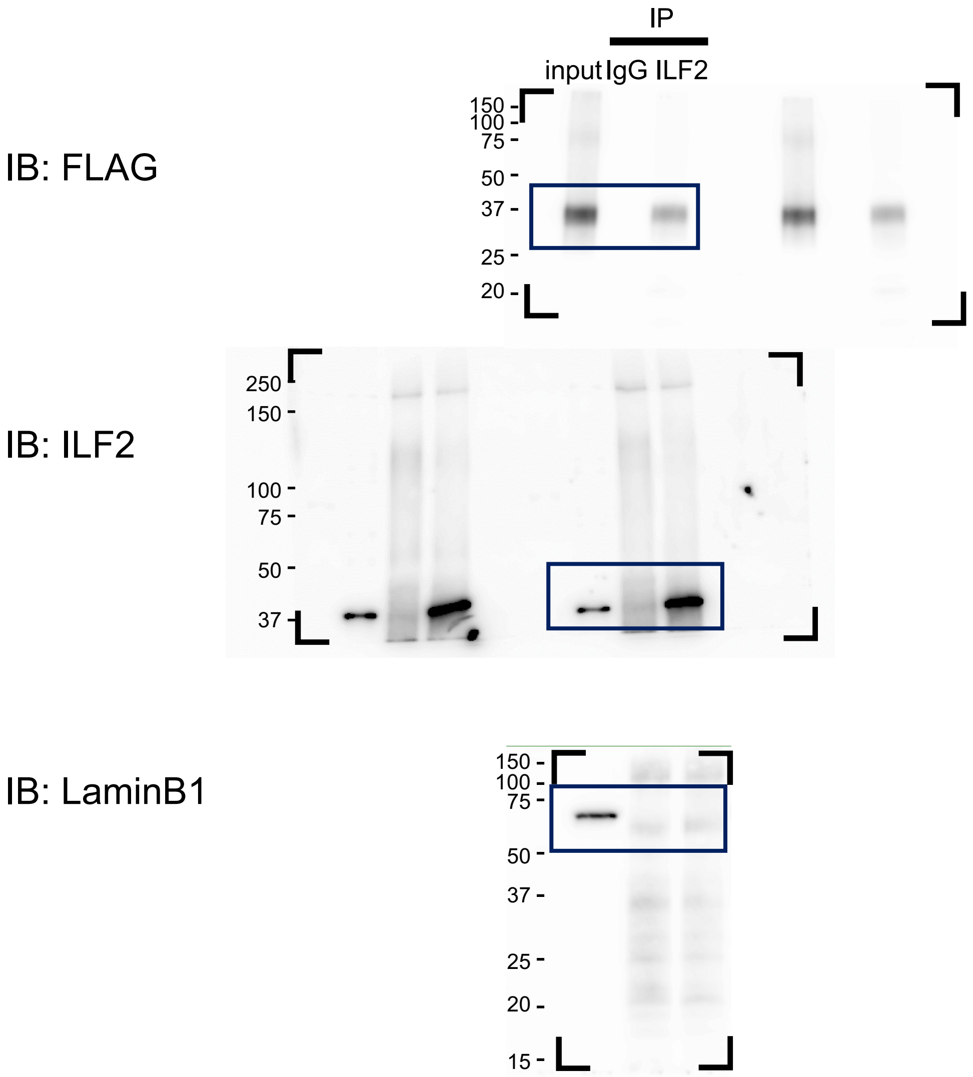

Supplementary Figure S18-3
Original blots for Supplementary Figure S10c

IB: FLAG

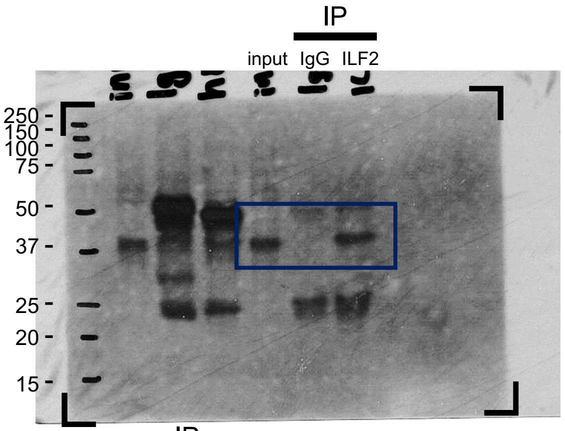

IB: ILF2

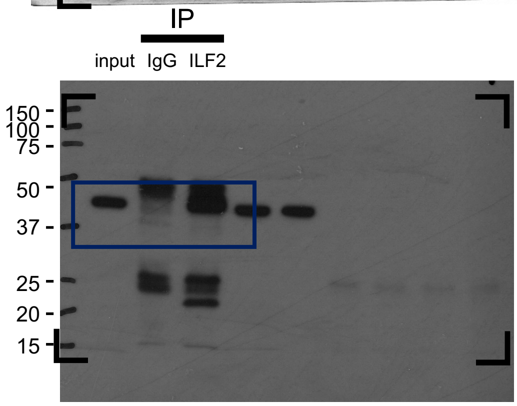

IB: Histon H3

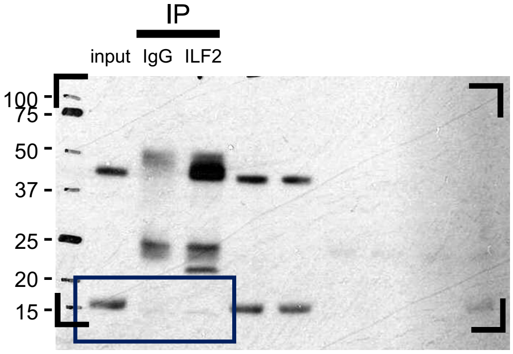

Supplementary Figure S19
Original gel for Supplementary Figure S12

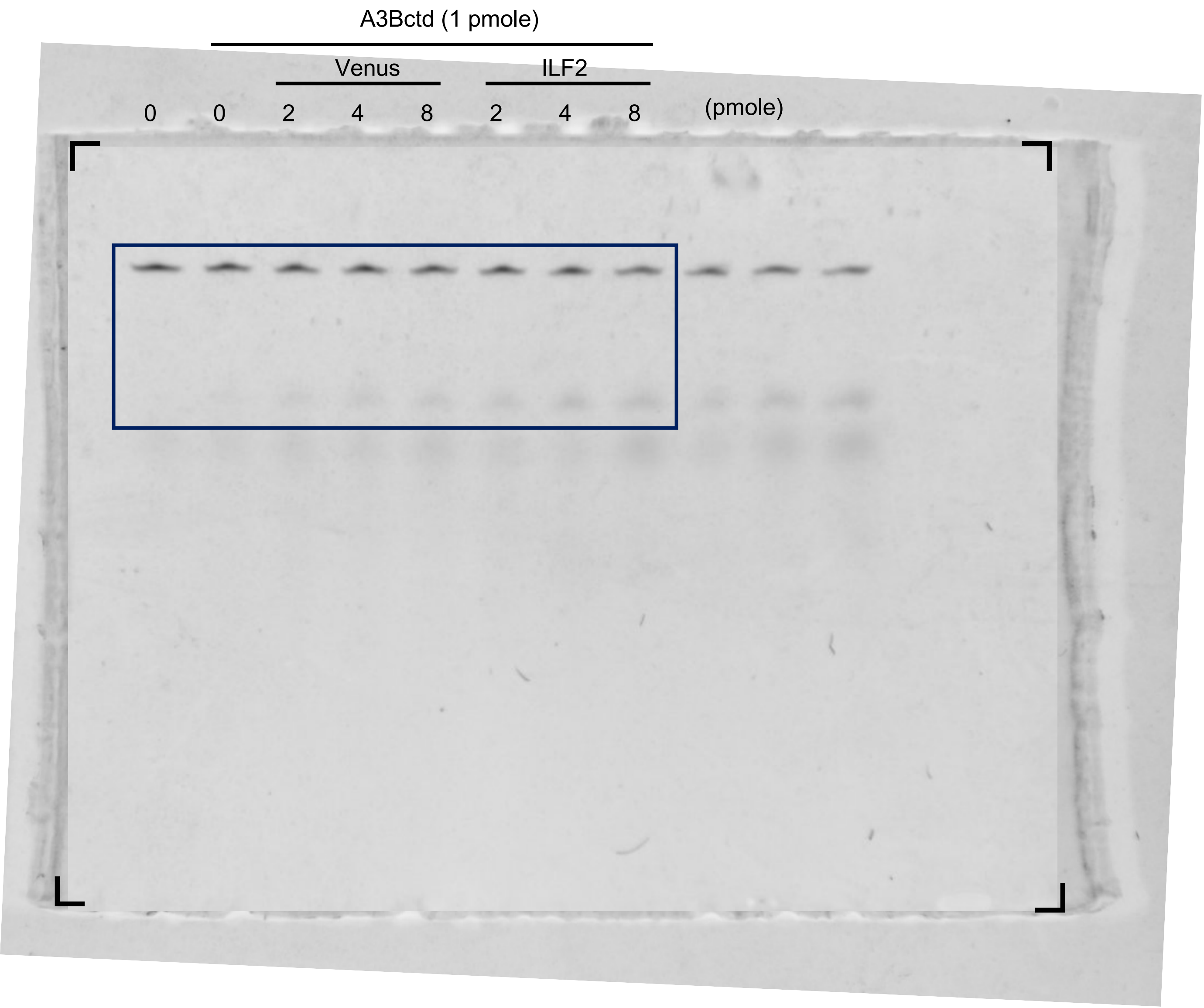

Legends for supplementary dataset files:

Supplementary dataset file 1

Raw data of the identified interacting protein candidates in AMO1-KI cells.

A3B interacting protein candidates in AMO1-KI cells are listed. Fold change of normalized relative abundance was calculated as the normalized abundance in AMO1-KI divided by that in AMO1-WT.

Supplementary dataset file 2

Raw data of the identified interacting protein candidates in RPMI-KI cells.

A3B interacting protein candidates in RPMI-KI cells are listed and fold change calculated in the same way as for AMO1-KI (Supplementary dataset file 1).

Supplementary dataset file 3

Gene ontology (GO) and pathway enrichment analyses of interacting proteins in AMO1-KI and RPMI-KI.

Significantly enriched GO Cellular Components, Biological Processes and Molecular Functions among the A3B-interacting proteins are ranked according to *p* values. The results of KEGG pathway analysis are also shown in Supplementary Figure S5a, b.
